# Antibiotics are associated with worse outcomes in lung cancer patients treated with chemotherapy and immunotherapy

**DOI:** 10.1038/s41698-024-00630-w

**Published:** 2024-07-16

**Authors:** Arielle Elkrief, Eder Orlando Méndez-Salazar, Jade Maillou, Chad M. Vanderbilt, Pooja Gogia, Antoine Desilets, Meriem Messaoudene, Daniel Kelly, Marc Ladanyi, Matthew D. Hellmann, Laurence Zitvogel, Charles M. Rudin, Bertrand Routy, Lisa Derosa, Adam J. Schoenfeld

**Affiliations:** 1 Department of Pathology, Memorial Sloan Kettering Cancer Center, New York, NY USA; 2 Human Oncology and Pathogenesis Program, Memorial Sloan Kettering Cancer Center, New York, NY USA; 3 Department of Medicine, Thoracic Oncology Service, Memorial Sloan Kettering Cancer Center, New York, NY USA; 4 University of Montreal Research Center (CR-CHUM), Montreal, QC Canada; 5 Department of Hematology-Oncology, Centre Hospitalier de l’Université de Montréal (CHUM), Montreal, QC Canada; 6 Informatics Systems, Memorial Sloan Kettering Cancer, New York, NY USA; 7 Department of Medicine, Weill Cornell Medical College, New York, NY USA; 8 INSERM U1015, Gustave Roussy Cancer Campus, Université Paris-Saclay, Villejuif, France

**Keywords:** Non-small-cell lung cancer, Prognostic markers

## Abstract

Anti-PD(L)-1 inhibition combined with platinum doublet chemotherapy (Chemo-IO) has become the most frequently used standard of care regimen in patients with non-small cell lung cancer (NSCLC). The negative impact of antibiotics on clinical outcomes prior to anti-PD(L)-1 inhibition monotherapy (IO) has been demonstrated in multiple studies, but the impact of antibiotic exposure prior to initiation of Chemo-IO is controversial. We assessed antibiotic exposures at two time windows: within 60 days prior to therapy (-60 d window) and within 60 days prior to therapy and 42 days after therapy (-60 + 42d window) in 2028 patients with advanced NSCLC treated with Chemo-IO and IO monotherapy focusing on objective response rate (ORR: rate of partial response and complete response), progression-free survival (PFS), and overall survival (OS). We also assessed impact of antibiotic exposure in an independent cohort of 53 patients. Univariable and multivariable analyses were conducted along with a meta-analysis from similar studies. For the -60 d window, in the Chemo-IO group (*N* = 769), 183 (24%) patients received antibiotics. Antibiotic exposure was associated with worse ORR (27% vs 40%, *p* = 0.001), shorter PFS (3.9 months vs. 5.9 months, hazard ratio [HR] 1.35, 95%CI 1.1,1.6, *p* = 0.0012), as well as shorter OS (10 months vs. 15 months, HR 1.50, 95%CI 1.2,1.8, *p* = 0.00014). After adjusting for known prognostic factors in NSCLC, antibiotic exposure was independently associated with worse PFS (HR 1.39, 95%CI 1.35,1.7, *p* = 0.002) and OS (HR 1.61, 95%CI 1.28,2.03, *p* < 0.001). Similar results were obtained in the -60 + 42d window, and also in an independent cohort. In a meta-analysis of patients with NSCLC treated with Chemo-IO (*N* = 4) or IO monotherapy (*N* = 13 studies) antibiotic exposure before treatment was associated with worse OS among all patients (*n* = 11,351) (HR 1.93, 95% CI 1.52, 2.45) and Chemo-IO-treated patients (*n* = 1201) (HR 1.54, 95% CI 1.28, 1.84). Thus, antibiotics exposure prior to Chemo-IO is common and associated with worse outcomes, even after adjusting for other factors. These results highlight the need to implement antibiotic stewardship in routine oncology practice.

## Introduction

Drugs that inhibit PD-1 or PD-L1 (PD-(L)1 blockade) alone (IO) or in combination with platinum-doublet chemotherapy (Chemo-IO) are now standard-of-care therapeutic regimens in patients with advanced non-small cell lung cancer (NSCLC) without a driver alteration^1–5^. Despite improvements in outcome with these therapies, primary and acquired resistance is still an important clinical challenge, and disease progression is common^6,7^. Moreover, current biomarkers of response such as PD-L1 or tumor mutational burden are suboptimal at predicting patient outcomes^8,9^. The gut microbiota has emerged as a clinically relevant biomarker of response in NSCLC^10,11^, with pre-clinical evidence demonstrating that specific commensals such as *Akkermansia muciniphila* were critical for response to IO in murine models. More recently, the presence of *A. muciniphila* was validated as a biomarker for IO benefit in a prospective clinical study^12^.

There is a growing body of evidence to suggest that antibiotic exposure prior to the initiation of IO disrupts the gut microbiota composition and negatively impacts the clinical outcomes of patients with NSCLC and other solid malignancies, even after adjusting for other adverse prognostic factors^10,13–16^. The largest meta-analysis thus far, examining antibiotic exposure prior to IO initiation across all solid tumors in 41,663 patients, demonstrated that antibiotics were negatively associated with survival^17,18^.

The relationship between antibiotic use and Chemo-IO efficacy is less clear. One study across 302 patients with NSCLC treated with Chemo-IO showed no difference in PFS between antibiotic-exposed and unexposed patients, but a trend towards worse OS in patients exposed to antibiotics (*p* = 0.05)^19^. The addition of chemotherapy to IO could already disrupt gut microbiota and potentially make the impact of antibiotic exposure less clinically significant^20^.

Given increasing use of Chemo-IO as a mainstay first-line therapy in patients with NSCLC, and recent approvals of Chemo-IO in the neoadjuvant setting ^21^, there is a need to further understand the role of antibiotic use in clinical outcomes prior to the initiation of Chemo-IO. Therefore, the objective of this study was to determine the association of antibiotic exposure on clinical outcomes prior to the initiation of Chemo-IO in NSCLC. As a comparator, we also examined the association of antibiotic exposure on clinical outcomes prior to the initiation of IO in NSCLC, and performed a meta-analysis across all available studies.

## Results

### Patients

A total of 2028 patients were included, with 769 in the Chemo-IO cohort and 1259 treated with IO monotherapy (Table 1 and Supplementary Fig. 1). In the Chemo-IO cohort, 183 (24%) were exposed to antibiotics within 60 days prior to the initiation of Chemo-IO. In the IO monotherapy cohort, 277 (22%) were exposed to antibiotics within 60 days prior to the initiation of IO monotherapy. Categories of antibiotics are present in Supplementary Table 1. The most common single-agent antibiotics were cephalosporins, sulfonamides, and quinolones. Combination regimens were also frequently used, with the breakdown of specific combinations presented in Supplementary Table 2. Routes of antibiotics prescription are presented in Supplementary Table 3. Younger patients were more likely to be exposed to antibiotics (*p* < 0.001) in both Chemo-IO and IO monotherapy cohorts (Table 1). In the IO monotherapy cohort, there was a higher proportion of patients with brain metastases at baseline in the antibiotic group (Table 1). Other baseline characteristics in the antibiotic-exposed vs non-exposed groups were similar (Table 1). Consistent with treatment guidelines and recent approval of Chemo-IO, most patients in this group were treated in the first-line setting, with similar distribution of line of therapy among antibiotic-exposed and unexposed groups.

**Table 1:** Baseline characteristics according to antibiotics exposure in the Chemo-IO cohort and IO monotherapy cohort

Characteristic	Chemo/IO			IO		
	No antibiotics, *N* = 586^a^	Antibiotics, *N* = 183^b^	*p*-value^b^	No Antibiotics, *N* = 982^a^	Antibiotics, *N* = 277^a^	*p*-value^c^
**Age**	69 (61, 76)	66 (60, 72)	<0.001	69 (62, 75)	67 (59, 73)	**<0.001**
**Sex**			0.3			0.056
F	275 (47%)	94 (51%)		514 (52%)	127 (46%)	
M	311 (53%)	89 (49%)		468 (48%)	150 (54%)	
**ECOG PS**			0.5			>0.9
>=2	80 (15%)	22 (13%)		105 (11%)	29 (10%)	
0-1	460 (85%)	148 (87%)		877 (89%)	248 (90%)	
Unknown	46	13				
**Histology**			>0.9			>0.9
Adenocarcinoma	454 (77%)	143 (78%)		728 (74%)	208 (75%)	
Other	51 (8.7%)	16 (8.7%)		77 (7.8%)	20 (7.2%)	
Squamous cell carcinoma	81 (14%)	24 (13%)		177 (18%)	49 (18%)	
**Smoking history**			0.7			0.9
Current/Former	504 (86%)	158 (87%)		862 (88%)	244 (88%)	
Never	80 (14%)	23 (13%)		120 (12%)	33 (12%)	
Unknown	2	2				
**PD-L1%**	0 (0, 5)	0 (0, 5)	0.4	15 (0, 75)	20 (0, 70)	0.8
Unknown	110	23		410	115	
**PDL1 status**			0.2			0.8
<1%	300 (62%)	109 (68%)		219 (38%)	58 (36%)	
>=50%	46 (9.5%)	17 (11%)		239 (42%)	72 (44%)	
1-49%	139 (29%)	35 (22%)		115 (20%)	32 (20%)	
Unknown	101	22		409	115	
**Line of therapy**			0.8			>0.9
≥2	14 (2.4%)	3 (1.6%)		669 (68%)	188 (68%)	
1	572 (98%)	180 (98%)		313 (32%)	89 (32%)	
**Baseline brain metastases**			0.7			**0.022**
Absence	432 (77%)	134 (76%)		283 (77%)	78 (66%)	
Presence	127 (23%)	43 (24%)		86 (23%)	40 (34%)	
Unknown	27	6		613	159	
**Baseline liver metastases**			0.3			0.7
Absence	492 (87%)	159 (90%)		279 (76%)	87 (74%)	
Presence	74 (13%)	18 (10%)		90 (24%)	31 (26%)	
Unknown	20	6		613	159	

^a^Median (IQR); *n* (%).

^b^Wilcoxon rank sum test; Pearson’s Chi-squared test; Fisher’s exact test.

^c^Wilcoxon rank sum test; Pearson’s Chi-squared test.

Bold values indicate statistical signficance.

### Antibiotics use is negatively associated with survival in patients with NSCLC treated with Chemo-IO

**Fig. 1:**
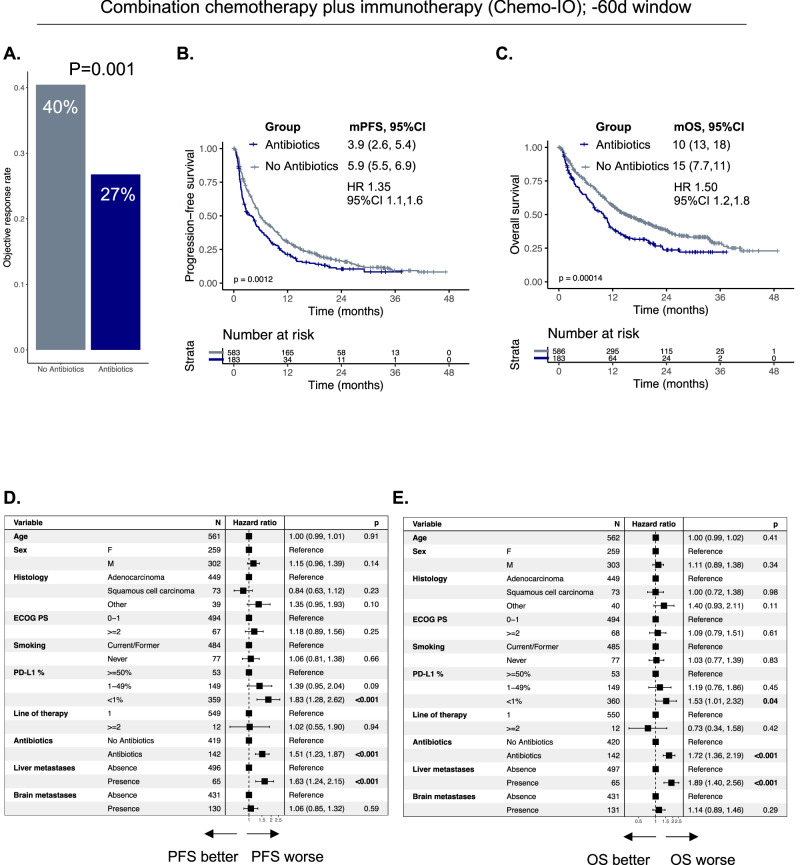
Association between antibiotic exposure and outcomes to combination platinum-doublet chemotherapy and immunotherapy in patients with non-small cell lung cancer. **A** Objective response rate, **B** Progression-free survival, **C** Overall survival in antibiotics vs. no antibiotics group. **D** Multivariable cox model for PFS and (**E**). OS for antibiotics vs. no antibiotics while adjusting for standard prognostic features in non-small cell lung cancer. mPFS, median progression-free survival; mOS, median overall survival; HR, hazard ratio; 95%CI, 95% confidence interval. Median survival times given and numbers in parentheses represent 95% confidence intervals.

**Fig. 2:**
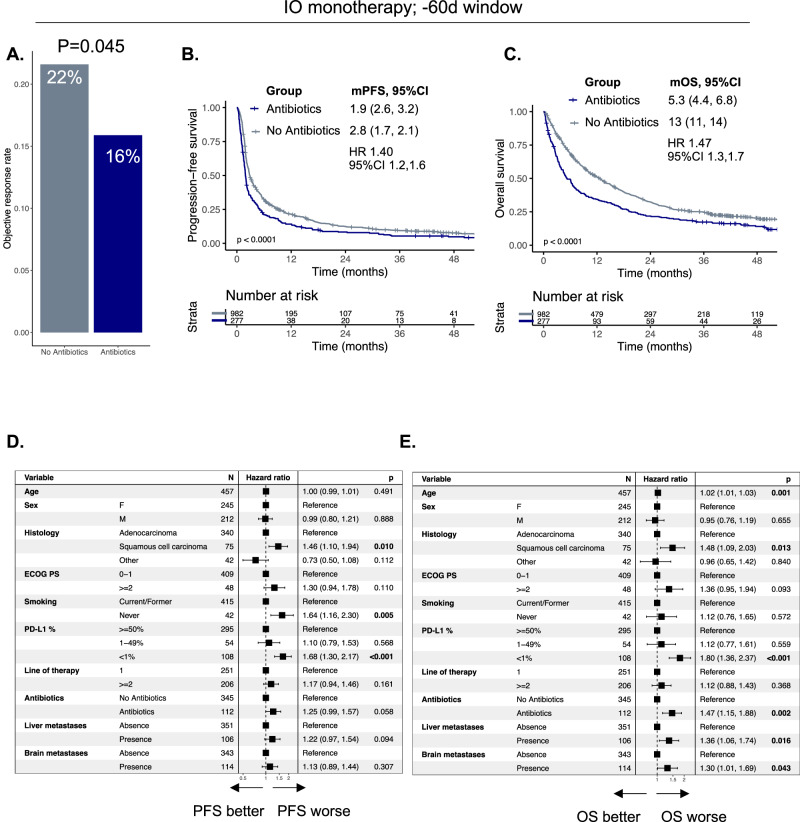
Association between antibiotic exposure and outcomes to immunotherapy alone in patients with non-small cell lung cancer. **A** Objective response rate, **B** Progression-free survival, **C** Overall survival in antibiotics vs. no antibiotics group. **D** Multivariable cox model for PFS and (**E**). OS for antibiotics vs. no antibiotics while adjusting for standard prognostic features in non-small cell lung cancer. mPFS, median progression-free survival; mOS, median overall survival; HR, hazard ratio; 95%CI, 95% confidence interval. Median survival times given and numbers in parentheses represent 95% confidence intervals.

We first explored the role of antibiotic exposure in the Chemo-IO cohort in the -60d window. Patients in the antibiotic group had lower ORR (27% vs 40%, *p* = 0.001) (Fig. 1A). Median PFS was significantly shorter in the antibiotic group (3.9 months vs. 5.9 months, HR 1.35, 95%1.10,1.60, *p* = 0.0012) (Fig. 1B). Median OS was also significantly shorter in the antibiotic group (10 months vs. 15 months, HR 1.50, 95%CI 1.20,1.80, *p* = 0.00014) (Fig. 1C). In multivariable analyses adjusting for known prognostic factors, antibiotic exposure was independently associated with worse PFS (HR 1.51, 95%CI 1.23,1.87, *p* < 0.001) (Fig. 1D) and OS (HR 1.72, 95%CI 1.36,2.19, *p* < 0.001) (Fig. 1E). We next explored whether antibiotic class was associated with differential outcomes. We chose to compare the most frequently used regimen categories in our cohort. Patient baseline characteristics according to antibiotic category are present in Supplementary Table 4. Patients exposed to combination antibiotic regimens (> 1 agent of antibiotics concurrently) had ORR of 20% not significant after multiple hypothesis testing when comparing with the other groups, (*p* = 0.39) (Supplementary Fig. 2A). Moreover, patients exposed to combination antibiotic regimens had the shortest PFS (1.9 months vs. 5.9 months, HR 1.87, 95%CI 1.38, 2.54, *p* < 0.001) (ementary Fig. 2B) and OS (6.1 months vs. 15 months, HR 2.24, 95%CI 1.61, 3.11, *p* < 0.001) (Supplementary Fig. 2C). There was no significant difference in ORR based on antibiotic route (*p* = 0.37) (Supplementary Fig. 2D). Compared to the no antibiotic group, patients exposed to oral antibiotics had the shortest PFS (median PFS of 2.8 months HR 1.52, 95%CI 1.15,2, *p* = 0.003 compared to no antibiotic group), followed by intravenous antibiotics (median PFS of 4.2 months, HR 1.27, 95%CI 1.02, 1.58, *p* = 0.032) (Supplementary Fig. S2E). OS was similarly inferior in both the oral (median OS 9.9 months, HR 1.58, 95%CI 1.16,2.16, *p* = 0.004) and intravenous (median OS 10.0 months, HR 1.43, 95%CI 1.13, 1.82, *p* = 0.003) antibiotic group compared to no antibiotic group (Supplementary Fig. 2F).

We next sought to determine whether antibiotic exposure was associated with worse outcomes in patients with PD-L1 < 1%, as IO monotherapy is not approved as first-line treatment in PD-L1 < 1% tumors and Chemo-IO is the primary front-line approach in this setting. Baseline characteristics for this subgroup are presented in Supplementary Table 5. Antibiotic exposure in this subgroup was strongly associated with worse ORR (21% vs 37%, *p* = 0.006) (Supplementary Fig. 3A), PFS (2.8 months vs 5.5 months, HR 1.4, 95%CI 1.11,1.77, *p* = 0.0044) (Supplementary Fig. 3B), and OS (8.5 months vs. 14 months, HR 1.59, 95%CI 1.22,2.07, *p* = 0.00047) (Supplementary Fig. 3C). Like the overall population, antibiotic exposure was independently associated with PFS (HR 1.61, 95%CI 1.24, 2.09, *p* < 0.001) (Supplementary Fig. 3D) and OS (HR 1.73, 95%CI 1.29,2.31, *p* < 0.001) (Supplementary Fig. 3E) after adjusting for known prognostic factors.

Lastly, we performed an analysis taking into consideration antibiotics use prescribed 60 days before Chemo-IO initiation and 42 days after Chemo-IO initiation, as this window was originally described in the first paper on the deleterious impact of antibiotics in 2018^10^. Baseline characteristics and antibiotic information for this analysis are presented in Supplementary Tables 6–7. Using this antibiotics time window we observed similar results for ORR (*p* < 0.001), PFS (HR 1.35 95%CI 1.15, 1.59) and OS (HR 1.58 95%CI 1.31, 1.90) as well after multivariable analysis (Supplementary Fig. 4D-E). Taken together, these results demonstrate that antibiotic exposure within 60 days prior to Chemo-IO initiation is associated with worse ORR, PFS, and OS, even after adjusting for known prognostic factors. This was also the case for the -60 + 42d window. We also explored the impact of antibiotics strictly after start of Chemo-IO. Interestingly, ORR was not affected (*p* = 0.86) (Supplementary Fig. 5A), and there were no differences in PFS (HR 0.91, 95%CI 0.66,1.26) (Supplementary Fig. 5B) or OS (0.87, 95%CI 0.6,1.28) (Supplementary Fig. 5C).

We next explored the impact of antibiotics use in a separate independent cohort of 53 patients with advanced NSCLC treated with Chemo-IO (Supplementary Table 8), where antibiotics use 60 days prior to the initiation of treatment were associated with worse OS (10.82 months vs not reached, *p* = 0.0196, HR 0.30 95%CI 0.07, 1.35) (Supplementary Fig. 6A). Similar results were also obtained for the -60 + 42d window (10.82 months vs not reached, *p* = 0.0496 HR 0.37 95%CI 0.10, 1.33) (Supplementary Fig. 6B).

### Antibiotics are negatively associated with survival in patients with NSCLC treated with IO monotherapy

Consistent with previously published findings in patients treated with IO monotherapy, ORR was lower in the antibiotic-exposed group (16% vs. 22%, *p* = 0.045) relative to the non-exposed group (Fig. 2A). Similarly, PFS (1.9 months vs. 2.8 months, HR 1.40, 95%CI 1.2,1.6, *p* < 0.0001) (Fig. 2B) and OS (5.3 months vs. 13 months, HR 1.47, 95%CI 1.3, 1.7, *p* < 0.0001) (Fig. 2C) were shorter in the antibiotic-exposed group vs the non-exposed group. Worse PFS and OS were confirmed after adjusting for known prognostic factors (HR 1.25, 95%CI 0.99,1.57, *p* = 0.058 and HR 1.47, 95%, CI 1.15,1.88, *p* = 0.002 respectively) (Fig. 2D, E). Data for antibiotic categories and route are presented in Supplementary Fig. 7 and were largely consistent with findings in the Chemo-IO cohort, however, patients exposed to combination antibiotic regimens in the IO cohort were also more likely to be treated with ≥ 2 line IO, with higher proportion of presence of baseline brain metastases (Supplementary Table 9). We performed similar analyses for the –60 + 42d window for the IO monotherapy cohort with similar findings (Supplementary Fig. 8).

### Meta-analysis of association of antibiotic exposure and outcome in patients with NSCLC

**Fig. 3:**
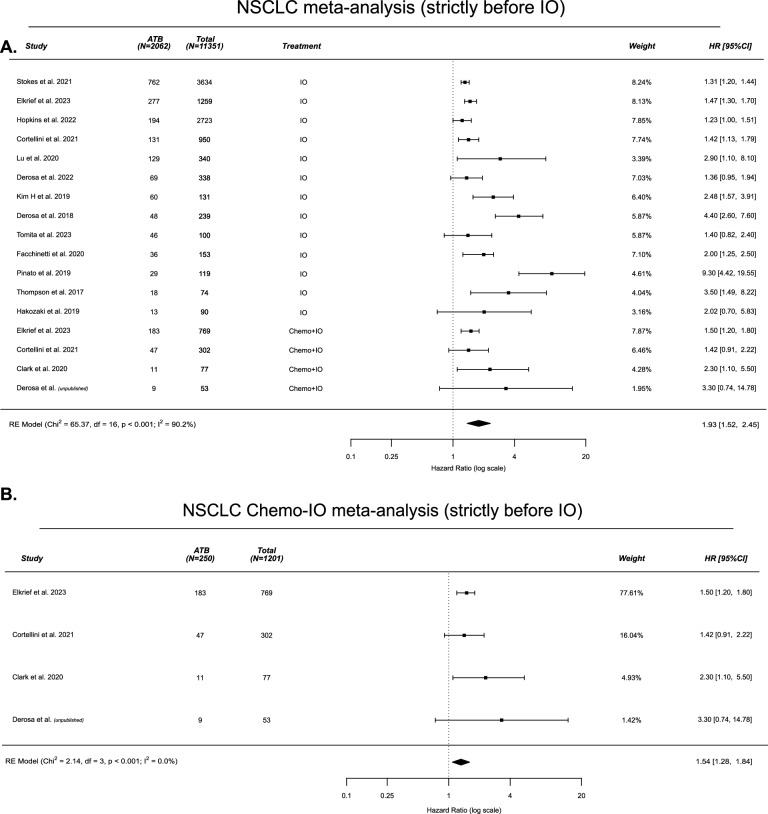
Meta-analysis of all studies examining the association of antibiotic exposure on overall survival to Immunotherapy (IO) alone or platinum doublet Chemo-IO in non-small cell lung cancer; strictly before initiation of IO or Chemo-IO. A literature review described in the **Methods** was performed to gather all available retrospective and prospective clinical studies evaluating the association between antibiotic exposure and outcome to either IO alone or Chemo-IO. Each included study is listed by row, with hazard ratios (HRs), study weightings (inverse variance), the total number of patients and the subset of antibiotic (ATB)-exposed patients denoted. RE model with representation of the *p*-value for heterogeneity between studies, random effects model; df, degrees of freedom. **A** For Chemo-IO and IO studies; **B** For Chemo-IO studies.

We next performed a meta-analysis of all available studies examining the association of antibiotic exposure on outcomes to IO or Chemo-IO in NSCLC, including data from the current study. Given the relatively large number of IO studies compared to Chemo-IO studies, we also performed a separate meta-analysis of including only the Chemo-IO studies. Supplementary Fig. 8 summarizes the studies included in the meta-analysis. As different studies used different time-windows, we separated the meta-analysis into studies evaluating impact of antibiotics strictly before therapy initiation or antibiotics before and during. Studies evaluating impact of antibiotics strictly after therapy initiation were excluded. First, in 11,351 patients with NSCLC with antibiotics exposure strictly before initiation of IO (*n* = 17 studies; *n* = 13 studies monotherapy IO and *n* = 4 studies Chemo-IO), antibiotics exposure was associated with significantly worse OS (HR 1.93, 95%CI 1.52-2.45) (Fig. 3A). Similar findings were observed when examining only the Chemo-IO studies in 1201 patients (HR 1.54, 95%CI 1.28-1.84) (Fig. 3B). In 12,220 patients with NSCLC with antibiotics exposure before initiation of IO and during IO (*n* = 40 studies), antibiotics exposure was also associated with worse OS (HR 1.57, 95%CI 1.38-1.78) (Supplementary Fig. 10). We extended this to a meta-analysis across all solid tumors (NSCLC, melanoma, genito-urinary and gastrointestinal malignancies) in 17,452 patients who received IO or Chemo-IO which demonstrated an association between antibiotics before IO and shorter OS (HR 1.98, 95%CI 1.72, 2.28) (Supplementary Fig. 11). This association was consistent if antibiotics were given before and during IO (HR 1.60, 95%CI 1.48, 1.73) (Supplementary Fig. 12). In a larger pool of patients (*n* = 46,232) with antibiotics given in the -90, + 90 window, antibiotics were also associated with worse outcome (HR 1.71, 95%CI 1.59, 1.83) (Supplementary Fig. 13). Taken together, these meta-analyses confirm previous data that antibiotic exposure prior to the initiation of IO as well as Chemo-IO is associated with worse OS, and we confirmed this specifically in patients with NSCLC treated with Chemo-IO.

## Discussion

In this study in 2028 patients with advanced NSCLC treated with IO-based regimens, receiving antibiotics was consistently associated with worse outcomes. Specifically, in the Chemo-IO group, antibiotic exposure was associated with worse ORR, PFS, and OS, even after adjusting for known prognostic factors. Patients who received >1 class of antibiotic at the same time had the worst outcomes, suggesting that broad-spectrum use may be associated with particularly poor outcomes. Patients receiving oral antibiotics had similarly worse outcomes to those treated with intravenous antibiotics; and both antibiotic route groups had worse outcomes compared to those not exposed to antibiotics, suggesting that route of administration may not be an important determinant of outcome. We validated the deleterious impact of antibiotics in patients receiving Chemo-IO in a meta-analysis of 1192 NSCLC patients.

Our study demonstrates worse outcomes associated with antibiotic exposure in patients treated with Chemo-IO, a primary first-line therapy in patients with stage IV NSCLC without sensitizing *EGFR* or *ALK* alterations. Our results suggest that similar practices and recommendations of antibiotic stewardship prior to IO monotherapy and combination of IO with chemotherapy are warranted^14^. While antibiotics are sometimes unavoidable, minimizing duration of exposure and selecting narrow-spectrum classes is advisable. Additionally, strategies to minimize the negative association of antibiotics on outcome, such as charcoal scavenger use^22^, merit clinical evaluation. Interestingly, the effect of cephalosporins was different in patients with received Chemo-IO compared to IO monotherapy. Future studies, including functional in vivo studies, will be required to understand the differential impact on antibiotic categories and outcome to IO.

Our study has multiple limitations including the potential confounding of poor performance status and infection in patients receiving antibiotics. However, the observation that antibiotic exposure was associated with worse outcome remained significant after adjusting for multiple factors including performance status, brain metastases, and liver metastases. In addition, we found that patients exposed to oral antibiotics compared to intravenous antibiotics had similarly worse outcomes compared to the no antibiotic group, suggesting that antibiotic exposure may not merely act as a surrogate for overall “sickness” or severity of infection. Nevertheless, we recognize the potential of co-linearity between antibiotics exposure and overall sickness. Despite adjustment for major prognostic factors in NSCLC, additional confounding by multiple other factors such as reason for antibiotic exposure and/other concomitant medication prescriptions such as steroids are additional limitations of this study. While our findings that antibiotics started strictly after the first cycle of Chemo-IO did not impact clinical outcomes, these findings were limited by small sample size and also did not adjust for potential impact of lead-time bias in these results. Lastly, another limitation of our study is unavailability of duration of antibiotics on outcome, as this data was not available.

Recently several microbiome studies from patients with cancer treated with ICI characterized antibiotics-related dysbiosis. These microbiome profiling studies identified that patients treated with antibiotics have a lower baseline diversity as well as downregulation of bacteria associated with IO response such as *Ruminococcus*^11,23,24^. Specifically, in a study of 70 Japanese patients with NSCLC, the impact of antibiotic exposure on the gut microbiome composition revealed that antibiotic use was associated with significantly decreased alpha diversity, with the global gut microbiome distribution demonstrating two separate clusters when comparing the antibiotic exposed and unexposed groups. Patients who did not receive antibiotic had enrichment of specifically *Ruminococcaceae* UCG 13, Clostridiales, and *Agathobacter*, whereas patients with antibiotic exposure had enrichment of *Hungatella*^11^, also associated with shorter OS in patients with NSCLC treated with IO^12^. Studies have also revealed that it can take >30 days for the microbiome to recover post-antibiotic therapy ^25^. Lastly, a large prospective study evaluated the impact of Akkermansia muciniphila on outcomes to second-line IO in 338 patients with NSCLC. Interestingly, the group with the highest percentile of *A. muciniphila* was associated with resistance to IO, and higher exposure to antibiotic prior to IO initiation^23^.

Antibiotics can alter the gut microbiome by inducing loss of diversity, loss of vital taxa, changes in gut microbiome metabolite profile, as well as decreasing colonization resistance against invasive pathogens inducing a change in intestinal barrier integrity, a key example of which is in the context of *C. difficile* infection^26^. However, until recently, the mechanism of antibiotic disruption of IO efficacy has remained elusive. Fidelle et al. identified in pre-clinical models that antibiotic-induced gut dysbiosis promoted downregulation of mucosal addressing cell adhesion molecule-1 (MAdCAM-1) in the ileum, which was accompanied by post-antibiotic gut recolonization with *Enterocloster* and *Hungatella*^16^. Downregulation of MAdCAM-1 led to exodus of immunosuppressive T cells and anti-PD-1 resistance. Altogether these results suggest a mechanism for poor response to IO in patients treated with antibiotics and a potential biomarker to detect antibiotics-related dysbiosis. In contrast, MAdCAM-1 levels were restored with fecal microbial transplantation. Moreover, in four independent cohorts of advanced lung (1/3 of which were treated with Chemo-IO combinations), kidney, and bladder cancer patients, low circulating levels of soluble MAdCAM-1 reflected gut dysbiosis and predicted resistance to PD-1 blockade^16^. These results suggest a mechanism for poor response to IO mediated by MAdCAM-1 in patients treated with antibiotics and a potential biomarker to detect antibiotics-related dysbiosis. This study also points to the potential clinical utility of restoring or improving the gut microbiota prior to initiation of therapy, for which there are several studies ongoing for fecal microbiota transplantation (NCT04951583), use of prebiotics (NCT05303493), or probiotics (NCT05094167).

In conclusion, we report the negative association of antibiotics on outcomes to Chemo-IO in patients with NSCLC. Our studies reinforce the need to minimize antibiotic exposure in the critical window prior to therapy initiation as well as the need to employ antibiotic stewardship and selection of narrow-spectrum antibiotics for shorter durations when possible.

## Methods

### Patients

#### MSK cohort

After institutional review board approval (16-1144) and according to the Declaration of Helsinki, patients with advanced NSCLC seen at Memorial Sloan Kettering Cancer Center [MSK] between 2011 and 2020 were assessed in this retrospective analysis. Informed consent was waved under the IRB approved retrospective research. Only patients with advanced NSCLC without sensitizing alterations in *EGFR* or *ALK* treated with IO monotherapy or Chemo-IO were eligible for analysis (Supplementary Fig. 1). Patients in the IO monotherapy and Chemo-IO cohorts were analyzed separately given known differences in baseline characteristics and clinical outcomes between these two groups^27^. Antibiotic exposure was extracted from electronic health records (EHRs) and defined as exposure to antibiotics within 60 days prior to initiation of therapy, consistent with other studies^28^. We also looked at an additional window of exposure with a wider time period between 60 days before and 42 days after initiation of therapy (-60 + 42d window) as initially published on the first paper describing the negative impact of antibiotics on IO^10^.

PD-L1 expression (tumor proportion score) was evaluated in patients with available tissue and reported as the percentage of tumor cells with membranous staining as previously described^29^.

#### Validation cohort

We also assessed the impact of antibiotics use in an independent validation cohort of 53 NSCLC patients treated with Chemo-IO. Clinical data collection was performed under the study ONCOBIOTICS* (Sponsor Protocol N: CSET 2017/2619, ID-RCB N: 2017-A02010-53) according to the ethical guidelines and approval of the local ethical committee (Comité Consultatif de Protection des Personnes dans la Recherche Biomédicale (CCPPRB) of the Kremlin Bicêtre Hospital) and according to the Declaration of Helsinki. ONCOBIOTICS is multicentric prospective observational study recruiting cancer patients with advanced NSCLC in France since 2017. Antibiotics exposure was defined in the same way as in the MSK cohort for the two windows.

### Statistical analysis

Descriptive statistics were used to describe the analysis population stratified by antibiotic exposure in both Chemo-IO and IO monotherapy groups. Differences in baseline characteristics by group were evaluated using the Wilcoxon rank sum test, Fisher’s exact test, or Pearson’s Chi-squared test as appropriate.

Patients who did not experience progression or death by the data lock were censored at date of last assessment. Investigator-assessed objective response rate (ORR) was defined as the rate of partial response plus complete response. Real-world investigator-assessed progression-free survival (PFS) was assessed from the date the patient began therapy to the date of progression as previously described^30–32^. Overall survival (OS) was calculated from treatment start date until date of death or last follow-up. Kaplan-Meier curves and log-rank test statistics were computed to compare PFS and OS between groups. Multivariable Cox regression models were used to determine hazard ratios (HRs) and 95% confidence intervals (CIs) for PFS and OS between antibiotic exposed and non-exposed groups, adjusting for clinicopathologic features including: age, sex, Eastern Cooperative Oncology Group (ECOG) performance status, histology, smoking history, line of treatment, presence/absence of brain and/or liver metastases, and PD-L1 expression. Statistical tests were two-sided and a *p*-value <0.05 was considered statistically significant. Analyses were conducted using R version 4.1.1 with the tidyverse (v1.3.1)^33^, gtsummary (v1.6.0)^34^, survival (v3.3.1) and survminer (v0.4.9) packages^35^.

### Meta-analysis

For the meta-analysis, a systematic literature search was performed using the following PubMed query: (immunotherapy[Title/Abstract] OR immunotherapies[Title/Abstract] OR ICI[Title/Abstract] OR ICIs[Title/Abstract] OR “immune checkpoint inhibitor”[Title/Abstract] OR “immune checkpoint inhibitors”[Title/Abstract] OR “immune-checkpoint inhibitors”[Title/Abstract] OR “immune-checkpoint inhibitor”[Title/Abstract] OR “PD-1”[Title/Abstract] OR PD1[Title/Abstract] OR PDL1[Title/Abstract] OR “PD-L1”[Title/Abstract]) AND (antibiotics[Title/Abstract] OR antibiotic[Title/Abstract]). In addition, major oncology conference proceedings, such as the American Society of Clinical Oncology (ASCO) and the European Society for Medical Oncology (ESMO), held between 2017 and 2024 were also screened to identify additional studies that could be incorporated provided they were not already included. To be included in our meta-analysis, studies had to meet the following criteria: (1) Studies published between 2017 and 2024; (2) Patients included in the study with a diagnosis of solid malignancy treated with IO, either as monotherapy or in combination with chemotherapy; (3) The antibiotic exposure occurred strictly within the defined timeframes (before or before and during IO), regardless of antibiotic class, route of administration, and duration of use; (4) Studies with evaluation of OS according to antibiotic exposure; and (5) Available HR statistic accompanied by a 95% confidence interval from univariate or adjusted Cox multivariable analysis.

From each of the eligible studies, the following data were collected: basic information of literature (author, publication year, type of publication), information on the population (number of patients included), cancer characteristics (type and stage of cancer); information on immunotherapy (type of IO and if combined with another therapy), information on antibiotic treatment (number of patients exposed to antibiotics, class of antibiotics, timing of antibiotics exposure relative to IO treatment initiation, and duration), and outcomes, including HR and 95% CIs for OS. In the absence of HR and 95% CIs for OS in numerical reports, data were estimated from descriptive graphs or calculated using the exponentially weighted average of the logarithmic HR, a general statistical method commonly used in meta-analyses.

The systematic search of PubMed using a comprehensive query resulted in 874 results that had been published between 2017 and 2024. Of these studies, 783 did not meet inclusion criteria, leaving a total of 91 studies that evaluated the association of antibiotics on the survival of patients with cancer treated with IO. Additionally, 43 relevant abstracts and posters were identified from 12 major oncology conference proceedings. A total of 31 studies were further excluded due the redundancy of data (abstract presented as a conference and published final article; when both were available the published article was selected), the hazard ratio for the OS either not being reported or being inaccessible, and the antibiotic exposure not being within the defined timeframe. Thus, 75 articles published between 2017 and 2024 in peer-reviewed journals, 28 posters and abstracts, the current study were ultimately included in the meta-analysis, representing a total of 105 studies. The log-transformed hazard ratio (HR) and its standard error were extracted from the published studies in order to calculate the summary effect size. We used a random-effects model with restricted maximum likelihood (REML) method to estimate the between-study variance component. The significance of the observed heterogeneity was performed with chi-squared test and the statistic I^2^ among the studies. Finally, the inverse variance method was used to calculate weights for each study. The resulting summary effect size and its 95% confidence interval were plotted on the forest plot for visual representation using the metafor R package.

### Reporting summary

Further information on research design is available in the Nature Research Reporting Summary linked to this article.

### Supplementary information

Supplemental Material

Reporting Summary

## Data Availability

All data will be made available upon reasonable request to the corresponding author.

## Code availability

All code will be made available upon reasonable request to the corresponding author.
