# Supplementary material for: Antibiotics are associated with worse outcomes in lung cancer patients treated with chemotherapy and immunotherapy
Source: NPJ Precis Oncol. 2024 Jul 16;8:143. doi: 10.1038/s41698-024-00630-w (PMC11252311; doi:10.1038/s41698-024-00630-w)
Supplement: Supplementary file 1 — Supplemental Material [file 41698_2024_630_MOESM1_ESM.pdf]

Table S1. Types of antibiotics -60d window

**Characteristic**	**N = 460**[1]
Antibiotic type	
combination regimen	191 (42%)
cephalosporins	121 (26%)
sulfonamides	55 (12%)
quinolones	33 (7.2%)
miscellaneous antibiotics	23 (5.0%)
macrolide derivatives	18 (3.9%)
penicillins	13 (2.8%)
tetracyclines	3 (0.7%)
glycopeptide antibiotic	2 (0.4%)
amebicides	1 (0.2%)
[1] n (%)	

Table S2. Breakdown of individual combination regimens used –60d window

**Characteristic**	**N = 191**[1]
cephalosporins | sulfonamides	27 (14%)
cephalosporins | macrolide	11(5.8%)
cephalosporins | quinolones	9 (4.7%)
macrolide | quinolones	9 (4.7%)
quinolones | sulfonamides	9 (4.7%)
cephalosporins | macrolide | penicillins | sulfonamides	6 (3.1%)
cephalosporins | quinolones | sulfonamides	5 (2.6%)
macrolide | sulfonamides	5 (2.6%)
cephalosporins | glycopeptide | penicillins	4 (2.1%)
miscellaneous | quinolones	4 (2.1%)
penicillins | quinolones	4 (2.1%)
cephalosporins | glycopeptide | miscellaneous | sulfonamides	3 (1.6%)
cephalosporins | macrolide | penicillins | quinolones	3 (1.6%)
cephalosporins | macrolide | sulfonamides	3 (1.6%)
cephalosporins | penicillins	3 (1.6%)
cephalosporins | penicillins | quinolones	3 (1.6%)
macrolide | penicillins | quinolones	3 (1.6%)
miscellaneous | penicillins	3 (1.6%)
penicillins | sulfonamides	3 (1.6%)
amebicides | cephalosporins | glycopeptide | macrolide | penicillins | sulfonamides	2 (1.0%)
amebicides | quinolones	2 (1.0%)
cephalosporins | glycopeptide | macrolide | penicillins | quinolones	2 (1.0%)
cephalosporins | glycopeptide | macrolide | penicillins | sulfonamides	2 (1.0%)
cephalosporins | macrolide | penicillins	2 (1.0%)
cephalosporins | macrolide | penicillins | quinolones | sulfonamides	2 (1.0%)
cephalosporins | miscellaneous | sulfonamides	2 (1.0%)
cephalosporins | penicillins | quinolones | sulfonamides	2 (1.0%)
cephalosporins | penicillins | sulfonamides	2 (1.0%)
cephalosporins | tetracyclines	2 (1.0%)
glycopeptide | macrolide | penicillins | quinolones	2 (1.0%)
glycopeptide | macrolide | penicillins | quinolones | sulfonamides	2 (1.0%)
glycopeptide | penicillins	2 (1.0%)
macrolide | penicillins	2 (1.0%)
macrolide | penicillins | quinolones | sulfonamides	2 (1.0%)
amebicides | aminoglycosides | cephalosporins | macrolide | miscellaneous | quinolones	1 (0.5%)
amebicides | carbapenems | cephalosporins | glycopeptide | penicillins | quinolones | sulfonamides	1 (0.5%)
amebicides | cephalosporins | glycopeptide | sulfonamides	1 (0.5%)
amebicides | cephalosporins | miscellaneous | penicillins | quinolones	1 (0.5%)
amebicides | cephalosporins | miscellaneous | sulfonamides	1 (0.5%)
amebicides | cephalosporins | penicillins | quinolones | sulfonamides	1 (0.5%)
amebicides | glycopeptide | miscellaneous | quinolones | sulfonamides	1 (0.5%)
amebicides | glycopeptide | penicillins	1 (0.5%)
amebicides | macrolide	1 (0.5%)
amebicides | penicillins | quinolones	1 (0.5%)
aminoglycosides | miscellaneous	1 (0.5%)
antituberculosis agents | macrolide | penicillins | quinolones	1 (0.5%)
carbapenems | cephalosporins | glycopeptide | macrolide | penicillins | quinolones	1 (0.5%)
carbapenems | cephalosporins | glycopeptide | macrolide | penicillins | sulfonamides	1 (0.5%)
carbapenems | glycopeptide | quinolones	1 (0.5%)
carbapenems | penicillins | quinolones	1 (0.5%)
carbapenems | penicillins | sulfonamides	1 (0.5%)
cephalosporins | glycopeptide	1 (0.5%)
cephalosporins | glycopeptide | macrolide | penicillins	1 (0.5%)
cephalosporins | glycopeptide | macrolide | penicillins | quinolones | sulfonamides	1 (0.5%)
cephalosporins | glycopeptide | miscellaneous	1 (0.5%)
cephalosporins | glycopeptide | penicillins | quinolones	1 (0.5%)

cephalosporins | glycopeptide | quinolones	1 (0.5%)
cephalosporins | glycopeptide | sulfonamides	1 (0.5%)
cephalosporins | macrolide | miscellaneous	1 (0.5%)
cephalosporins | miscellaneous	1 (0.5%)
cephalosporins | miscellaneous | quinolones	1 (0.5%)
cephalosporins | penicillins | quinolones | sulfonamides | tetracyclines	1 (0.5%)
glycopeptide | macrolide | penicillins | sulfonamides	1 (0.5%)
glycopeptide | miscellaneous	1 (0.5%)
glycopeptide | miscellaneous | quinolones | sulfonamides	1 (0.5%)
glycopeptide | miscellaneous | sulfonamides	1 (0.5%)
glycopeptide | penicillins | quinolones	1 (0.5%)
glycopeptide | penicillins | sulfonamides	1 (0.5%)
glycopeptide | quinolones | sulfonamides	1 (0.5%)
glycopeptide | sulfonamides	1 (0.5%)
macrolide | miscellaneous | sulfonamides	1 (0.5%)
macrolide | penicillins | sulfonamides	1 (0.5%)
macrolide | penicillins | sulfonamides | tetracyclines	1 (0.5%)
macrolide | quinolones | tetracyclines	1 (0.5%)
miscellaneous | sulfonamides	1 (0.5%)
penicillins | quinolones | sulfonamides	1 (0.5%)
quinolones | sulfonamides | tetracyclines	1 (0.5%)
sulfonamides | tetracyclines	1 (0.5%)
[1] n (%)	

Table S3. Route of antibiotics –60d window

Characteristic	N = 460[1]
Route	
Oral	260 (57%)
Intravenous	199 (43%)
Inhalation	1 (0.2%)

[1] n (%)

Table S4. Baseline characteristics according to Antibiotic category, Chemo-IO, –60d window

Characteristic	**No Antibiotics**, N = 586[1]	**cephalosporins**, N = 73[1]	**sulfonamides**, N = 32[1]	**combination regimen**, N = 50[1]	**p-value**[2]
**Age**	69 (61, 76)	66 (59, 73)	64 (59, 73)	65 (61, 70)	**0.003**
**Sex**					0.5
F	275 (47%)	36 (49%)	19 (59%)	22 (44%)	
M	311 (53%)	37 (51%)	13 (41%)	28 (56%)	
**ECOG PS**					0.10
>=2	80 (15%)	4 (6.2%)	5 (16%)	10 (22%)	
0-1	460 (85%)	61 (94%)	26 (84%)	36 (78%)	
Unknown	46	8	1	4	
**Histology**					0.8
Adenocarcinoma	454 (77%)	60 (82%)	26 (81%)	38 (76%)	
Other	51 (8.7%)	7 (9.6%)	1 (3.1%)	5 (10%)	
Squamous cell carcinoma	81 (14%)	6 (8.2%)	5 (16%)	7 (14%)	
**Smoking history**					0.3
Current/Former	504 (86%)	62 (85%)	25 (81%)	47 (94%)	
Never	80 (14%)	11 (15%)	6 (19%)	3 (6.0%)	
Unknown	2	0	1	0	
**Percent PDL1**					0.2
<1	300 (62%)	44 (68%)	18 (67%)	34 (74%)	
>=50	46 (9.5%)	5 (7.7%)	5 (19%)	5 (11%)	
1-49	139 (29%)	16 (25%)	4 (15%)	7 (15%)	
Unknown	101	8	5	4	
**Line of therapy**					0.7
>=2	14 (2.4%)	0 (0%)	0 (0%)	1 (2.0%)	
1	572 (98%)	73 (100%)	32 (100%)	49 (98%)	
**Baseline brain metastases**					**<0.001**
Absence	432 (77%)	62 (86%)	14 (45%)	37 (77%)	
Presence	127 (23%)	10 (14%)	17 (55%)	11 (23%)	
Unknown	27	1	1	2	
**Baseline liver metastases**					0.2
Absence	492 (87%)	64 (89%)	25 (81%)	46 (96%)	
Presence	74 (13%)	8 (11%)	6 (19%)	2 (4.2%)	
Unknown	20	1	1	2	

[1] Median (IQR); n (%)

[2] Kruskal-Wallis rank sum test; Pearson's Chi-squared test; Fisher's exact test

Table S5. Baseline characteristics for PDL1 <1% subgroup –60d window

Characteristic	Chemo/IO		
	**No Antibiotics**, N = 300[1]	**Antibiotics**, N = 109[1]	**p-value**[2]
**Age**	69 (62, 76)	65 (58, 72)	**<0.001**
**Sex**			0.8
F	142 (47%)	50 (46%)	
M	158 (53%)	59 (54%)	
**ECOG PS**			0.5
>=2	28 (10%)	13 (13%)	
0-1	249 (90%)	89 (87%)	
Unknown	23	7	
**Histology**			0.7
Adenocarcinoma	242 (81%)	84 (77%)	
Other	21 (7.0%)	9 (8.3%)	
Squamous cell carcinoma	37 (12%)	16 (15%)	
**Smoking history**			>0.9
Current/Former	261 (87%)	94 (87%)	
Never	38 (13%)	14 (13%)	
Unknown	1	1	
**percent_pd_l1**	0 (0%)	0 (0%)	
**PDL1 status**			
<1	300 (100%)	109 (100%)	
**Line of therapy**			0.3
>=2	6 (2.0%)	0 (0%)	
1	294 (98%)	109 (100%)	
**Baseline brain metastases**			>0.9
Absence	217 (76%)	78 (75%)	
Presence	70 (24%)	26 (25%)	
Unknown	13	5	
**Baseline liver metastases**			**0.035**
Absence	251 (87%)	98 (94%)	
Presence	39 (13%)	6 (5.8%)	
Unknown	10	5	

[1] Median (IQR); n (%)

[2] Wilcoxon rank sum test; Pearson's Chi-squared test; Fisher's exact test

Table S6. Baseline characteristics -60+42d window

Characteristic	Chemo/IO			IO		
	**No Antibiotics**, N = 491[1]	**Antibiotics**, N = 278[1]	**p-value**[2]	**No Antibiotics**, N = 835[1]	**Antibiotics**, N = 424[1]	**p-value**[2]
**Age**	69 (62, 76)	67 (61, 73)	0.006	69 (62, 75)	67 (59, 73)	**<0.001**
**Sex**			>0.9			0.6
F	236 (48%)	133 (48%)		429 (51%)	212 (50%)	
M	255 (52%)	145 (52%)		406 (49%)	212 (50%)	
**ECOG PS**			>0.9			0.7
>=2	65 (14%)	37 (14%)		87 (10%)	47 (11%)	
0-1	387 (86%)	221 (86%)		748 (90%)	377 (89%)	
Unknown	39	20				
**Histology**			0.088			0.8
Adenocarcinoma	373 (76%)	224 (81%)		617 (74%)	319 (75%)	
Other	51 (10%)	16 (5.8%)		67 (8.0%)	30 (7.1%)	
Squamous cell carcinoma	67 (14%)	38 (14%)		151 (18%)	75 (18%)	
**Smoking history**			0.2			0.084
Current/Former	417 (85%)	245 (89%)		743 (89%)	363 (86%)	
Never	72 (15%)	31 (11%)		92 (11%)	61 (14%)	
Unknown	2	2				
**percent_pd_l1**	0 (0, 10)	0 (0, 4)	0.071	15 (0, 75)	15 (0, 70)	0.5
Unknown	94	39		354	171	
**PDL1 status**			0.11			0.8
<1	244 (60%)	165 (68%)		178 (37%)	99 (39%)	
>=50	42 (10%)	21 (8.7%)		205 (43%)	106 (42%)	
Jan-49	119 (29%)	55 (23%)		99 (21%)	48 (19%)	
Unknown	86	37		353	171	
**Line of therapy**			0.6			0.4
>=2	12 (2.4%)	5 (1.8%)		562 (67%)	295 (70%)	
1	479 (98%)	273 (98%)		273 (33%)	129 (30%)	
**Baseline brain metastases**			0.9			**0.038**
Absence	364 (77%)	202 (77%)		238 (77%)	123 (69%)	
Presence	108 (23%)	62 (23%)		70 (23%)	56 (31%)	
Unknown	19	14		527	245	
**Baseline liver metastases**			0.3			0.2
Absence	413 (87%)	238 (89%)		238 (77%)	128 (72%)	
Presence	64 (13%)	28 (11%)		70 (23%)	51 (28%)	
Unknown	14	12		527	245	
[1] Median (IQR); n (%)						
[2] Wilcoxon rank sum test; Pearson's Chi-squared test						

Table S7. Types of antibiotics -60+42d window

Characteristic	N = 702[1]
Antibiotic category	
combination regimen	453 (65%)
cephalosporins	88 (13%)
sulfonamides	53 (7.5%)
quinolones	44 (6.3%)
macrolide	25 (3.6%)
miscellaneous	16 (2.3%)
penicillins	16 (2.3%)
tetracyclines	5 (0.7%)
glycopeptide	2 (0.3%)
[1] n (%)	

Table S8. Baseline characteristics for the IMMUNOLIFE cohort

Characteristic	Chemo-IO -60d window		
	No Antibiotics, N=44	Antibiotics -60, N=9	p-value
**Age**	67 (40,90)	66 (41,76)	0.4653
**Sex**			
F	9 (21%)	3 (33%)	0.6054
M	35 (79%)	6 (67%)	
**ECOG PS**			
>=2	6 (14%)	2 (22%)	0.6109
0-1	38 (86%)	7 (78%)	
Unknown	0	0	
**Histology**			
Adenocarcinoma	27 (61%)	7 (78%)	0.6442
Other	8 (13%)	1 (11%)	
Squamous cell carcinoma	9 (20)	1 (11%)	
**Smoking history**			
Current/Former	38 (93%)	8 (100%)	0.4297
Never	3 (7%)	0	
Unknown	3	1	
**Percent PDL1**	0 (0,100)	22,5 (0,60)	0.3891
Unknown	2	0	
**Line of therapy**			
>=2	0	0	1
1	44 (100%)	9 (100%)	
**Reason for ATB**			
Infection		7 (78%)	
Prophylaxis		2 (22%)	

Characteristic	Chemo-IO -60+42d window		
	No Antibiotics, N=40	Antibiotics -60+42, N=13	p-value
**Age**	67 (44,90)	66 (41,76)	0.4779
**Sex**			
F	8 (20%)	4 (31%)	0.6497
M	32 (80%)	9 (69%)	
**ECOG PS**			
>=2	6 (15%)	3 (23%)	0.6738
0-1	34 (85%)	10 (77%)	
Unknown	0	0	
**Histology**			

Adenocarcinoma	25 (63%)	9 (69%)	0.4524
Other	6 (15%)	3 (23%)	
Squamous cell carcinoma	9 (22%)	1 (8%)	
**Smoking history**			
Current/Former	34 (92%)	12 (100%)	0.5539
Never	3 (7%)	0	
Unknown	3	1	
**Percent PDL1**	0 (0,90)	5 (0,100)	0.3246
Unknown	2	0	
**Line of therapy**			
>=2	0	0	1
1	40 (100%)	13 (100%)	
**Reason for ATB**			
Infection		11 (69%)	
Prophylaxis		4 (31%)	

Table S9. Baseline characteristics according to Antibiotic category, IO monotherapy, -60d window

Characteristic	**No Antibiotics**, N = 982[1]	**cephalosporins**, N = 48[1]	**sulfonamides**, N = 23[1]	**combination regimen**, N = 141[1]	**p-value**[2]
**Age**	69 (62, 75)	67 (59, 71)	67 (62, 73)	66 (56, 71)	**<0.001**
**Sex**					0.3
F	514 (52%)	19 (40%)	12 (52%)	67 (48%)	
M	468 (48%)	29 (60%)	11 (48%)	74 (52%)	
**ECOG PS**					0.8
>=2	105 (11%)	6 (13%)	1 (4.3%)	17 (12%)	
0-1	877 (89%)	42 (88%)	22 (96%)	124 (88%)	
**Histology**					0.9
Adenocarcinoma	728 (74%)	38 (79%)	16 (70%)	102 (72%)	
Other	77 (7.8%)	2 (4.2%)	3 (13%)	11 (7.8%)	
Squamous cell carcinoma	177 (18%)	8 (17%)	4 (17%)	28 (20%)	
**Smoking history**					>0.9
Current/Former	862 (88%)	42 (88%)	20 (87%)	123 (87%)	
Never	120 (12%)	6 (13%)	3 (13%)	18 (13%)	
**Percent PDL1**					0.7
<1	219 (38%)	13 (32%)	3 (23%)	30 (40%)	
>=50	239 (42%)	22 (54%)	8 (62%)	32 (43%)	
Jan-49	115 (20%)	6 (15%)	2 (15%)	13 (17%)	
Unknown	409	7	10	66	
**Line of therapy**					**<0.001**
>=2	669 (68%)	22 (46%)	11 (48%)	109 (77%)	
1	313 (32%)	26 (54%)	12 (52%)	32 (23%)	
**Baseline brain metastases**					**0.014**
Absence	283 (77%)	25 (76%)	4 (40%)	34 (63%)	
Presence	86 (23%)	8 (24%)	6 (60%)	20 (37%)	
Unknown	613	15	13	87	
**Baseline liver metastases**					0.6
Absence	279 (76%)	26 (79%)	9 (90%)	38 (70%)	
Presence	90 (24%)	7 (21%)	1 (10%)	16 (30%)	
Unknown	613	15	13	87	
[1] Median (IQR); n (%)					
[2] Kruskal-Wallis rank sum test; Pearson's Chi-squared test; Fisher's exact test					

Supplementary Figure 1

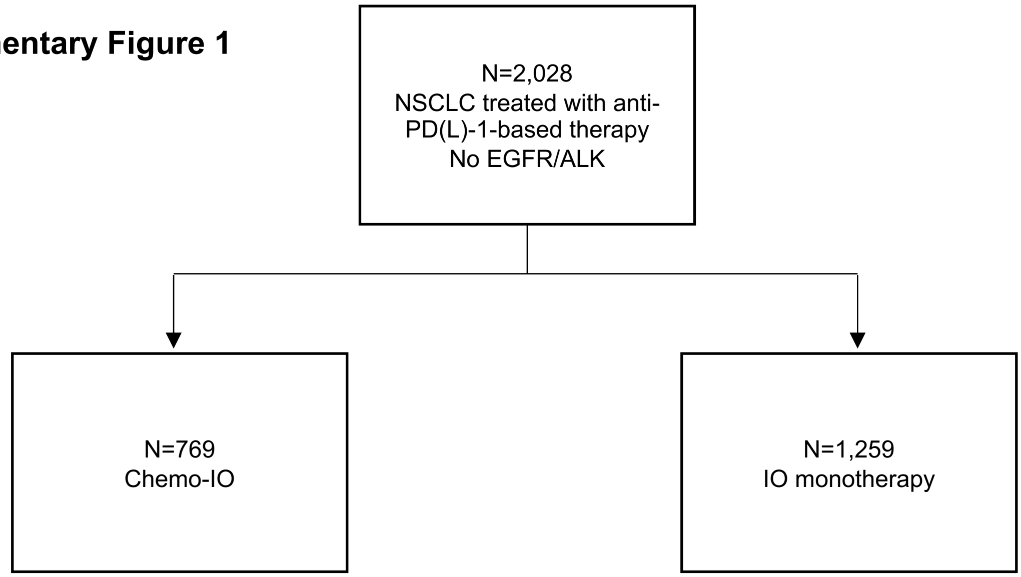

Supplementary Figure 1. Cohort Assembly.

Supplementary Figure 2

Combination chemotherapy plus immunotherapy (Chemo-IO), -60d window

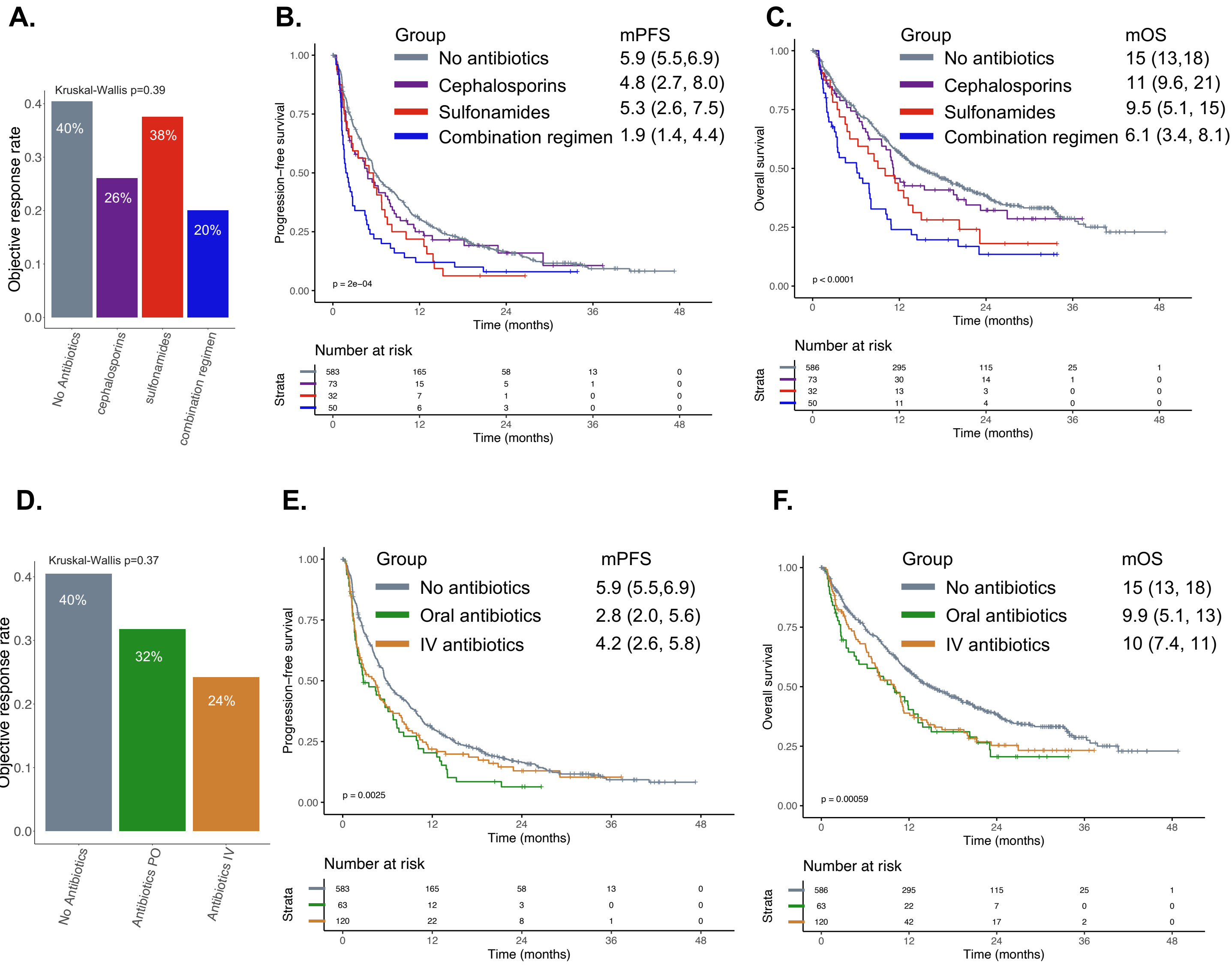

Supplementary Figure 2. Association between antibiotic class and route of administration with outcomes to combination Chemotherapy-immunotherapy in patients with non-small cell lung cancer. A. Objective response rate, **B.** Progression-free survival, **C.** Overall survival according to antibiotic class. **D.** Objective response rate, **E.** Progression-free survival, **F.** Overall survival according to antibiotic route. mPFS, median progression-free survival; mOS, median overall survival; HR, hazard ratio; 95%CI, 95 % confidence interval. Median survival times given and numbers in parentheses represent 95% confidence intervals.

Supplementary Figure 3

Chemo-IO, PD-L1<1%, (-60d window)

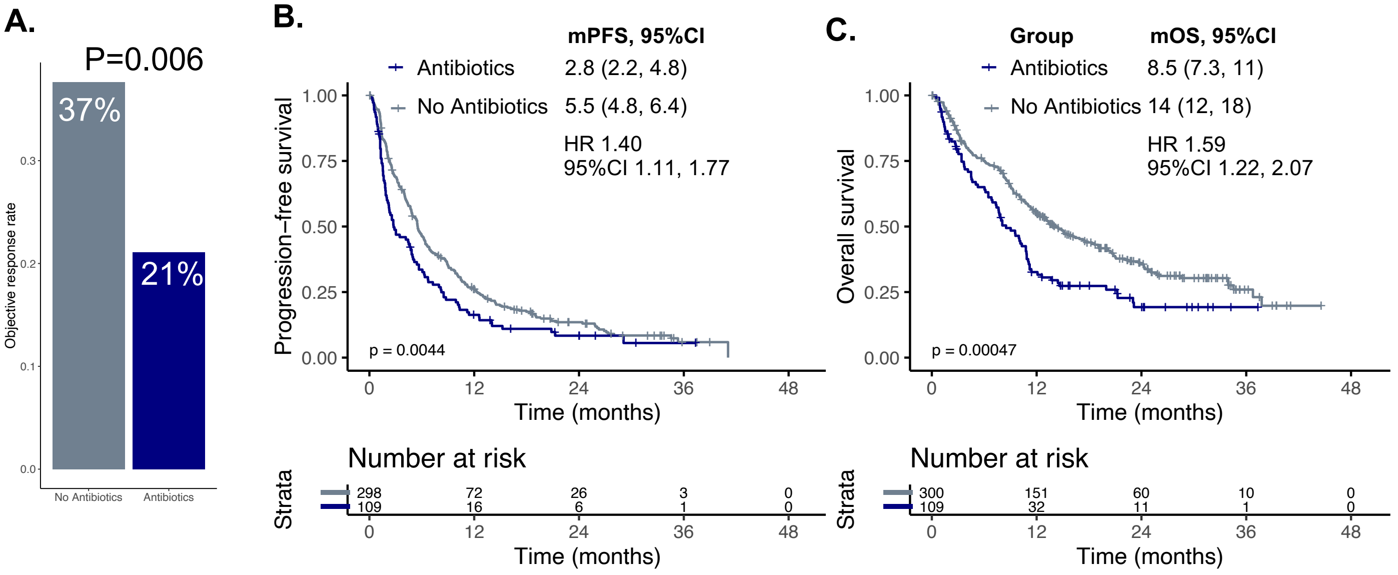

D.

Variable		N	Hazard ratio		p
**Age**		359		0.99 (0.98, 1.00)	0.060
**Sex**	F	163		Reference	
	M	196		1.16 (0.92, 1.45)	0.209
**Histology**	Adenocarcinoma	286		Reference	
	Squamous cell carcinoma	49		0.84 (0.59, 1.18)	0.306
	Other	24		1.32 (0.84, 2.08)	0.229
**ECOG PS**	0–1	320		Reference	
	>=2	39		1.14 (0.79, 1.65)	0.474
**Smoking**	Current/Former	313		Reference	
	Never	46		1.13 (0.81, 1.57)	0.469
**Line of therapy**	1	353		Reference	
	>=2	6		1.05 (0.46, 2.40)	0.908
**Antibiotics**	No Antibiotics	263		Reference	
	Antibiotics	96		1.61 (1.24, 2.09)	**<0.001**
**Liver metastases**	Absence	317		Reference	
	Presence	42		1.76 (1.24, 2.50)	**0.002**
**Brain metastases**	Absence	271		Reference	
	Presence	88		1.07 (0.82, 1.39)	0.634

0.5 1 1.5 2

PFS better PFS worse

E.

Variable		N	Hazard ratio		p
**Age**		360		1.00 (0.99, 1.01)	0.839
**Sex**	F	163		Reference	
	M	197		1.08 (0.83, 1.40)	0.583
**Histology**	Adenocarcinoma	286		Reference	
	Squamous cell carcinoma	49		0.98 (0.66, 1.44)	0.905
	Other	25		1.63 (1.00, 2.66)	0.051
**ECOG PS**	0–1	320		Reference	
	>=2	40		1.17 (0.78, 1.78)	0.444
**Smoking**	Current/Former	314		Reference	
	Never	46		1.05 (0.72, 1.52)	0.811
**Line of therapy**	1	354		Reference	
	>=2	6		0.66 (0.24, 1.84)	0.427
**Antibiotics**	No Antibiotics	264		Reference	
	Antibiotics	96		1.73 (1.29, 2.31)	**<0.001**
**Liver metastases**	Absence	318		Reference	
	Presence	42		1.81 (1.23, 2.67)	**0.003**
**Brain metastases**	Absence	271		Reference	
	Presence	89		1.14 (0.85, 1.54)	0.383

0.5 1 2

OS better OS worse

Supplementary Figure 3. Association between antibiotic exposure and outcomes to chemotherapy-immunotherapy in patients with non-small cell lung cancer with tumor PD-L1 <1%. A. Objective response rate, **B.** Progression-free survival, **C.** Overall survival according to antibiotic exposure. mPFS, median progression-free survival; mOS, median overall survival; HR, hazard ratio; 95%CI, 95 % confidence interval. Median survival times given and numbers in parentheses represent 95% confidence intervals.

Supplementary Figure 4

Chemo-IO, -60+42d window

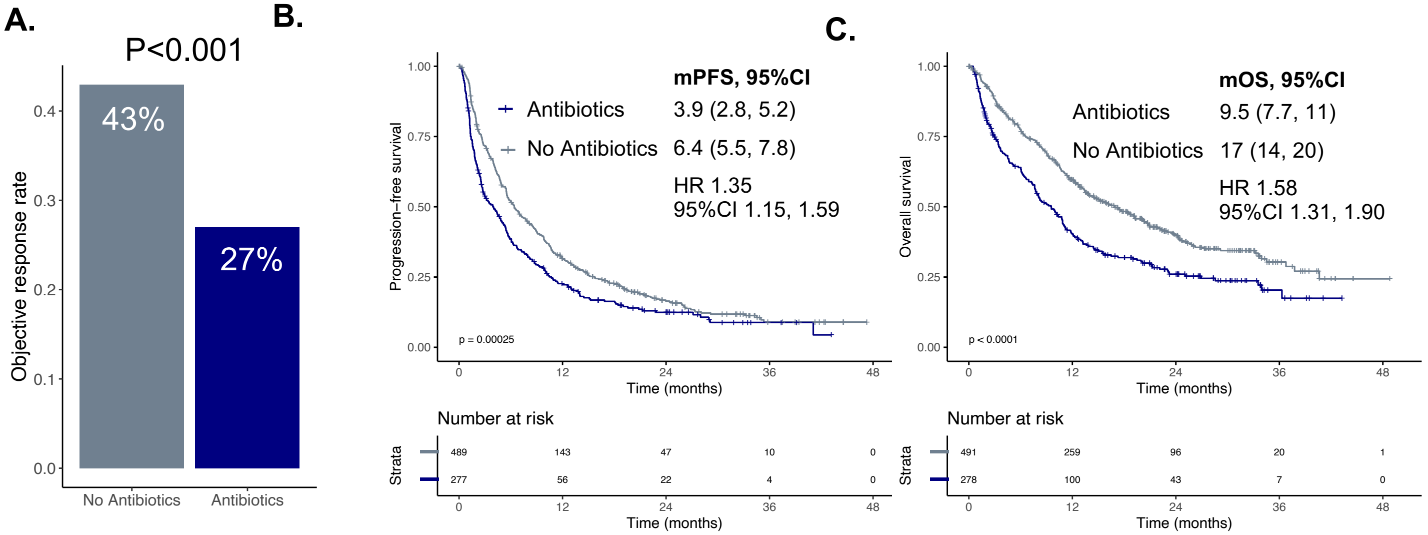

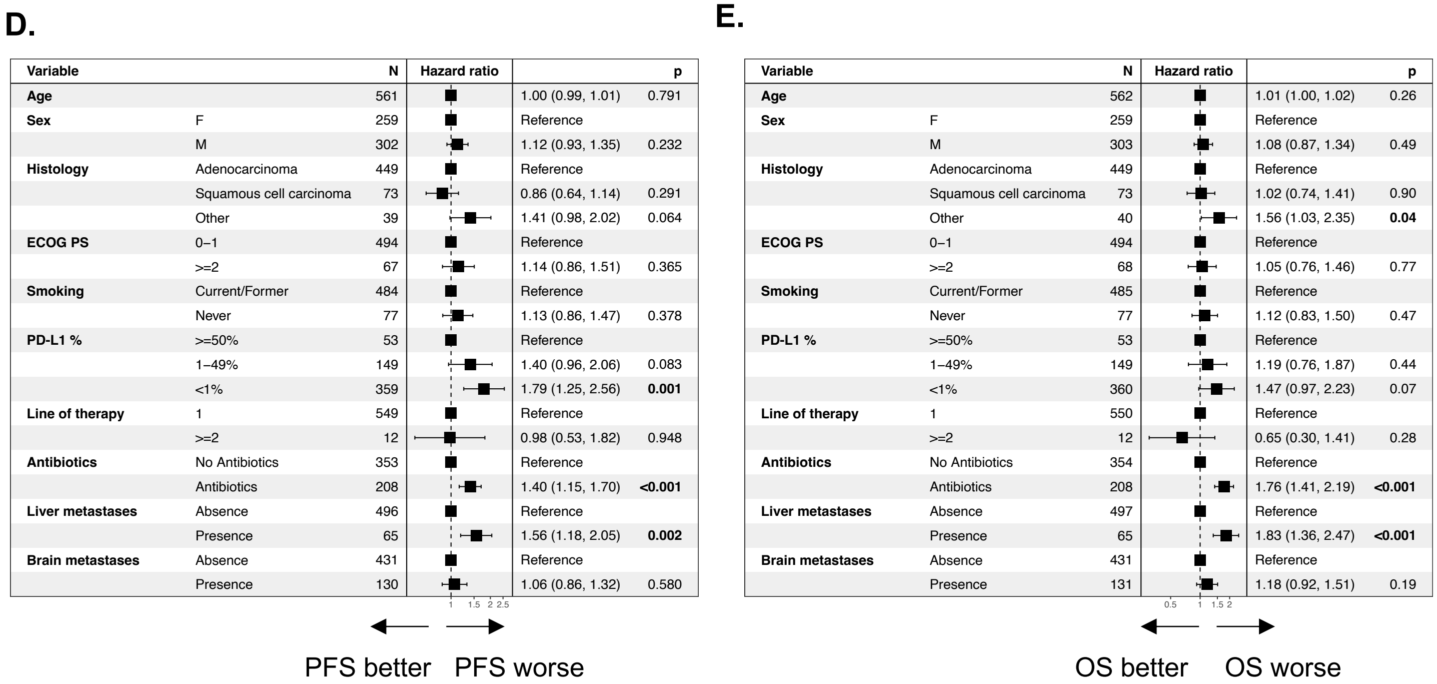

D.

Variable		N	p
**Age**		561	1.00 (0.99, 1.01) 0.791
**Sex**	F	259	Reference
	M	302	1.12 (0.93, 1.35) 0.232
**Histology**	Adenocarcinoma	449	Reference
	Squamous cell carcinoma	73	0.86 (0.64, 1.14) 0.291
	Other	39	1.41 (0.98, 2.02) 0.064
**ECOG PS**	0–1	494	Reference
	>=2	67	1.14 (0.86, 1.51) 0.365
**Smoking**	Current/Former	484	Reference
	Never	77	1.13 (0.86, 1.47) 0.378
**PD-L1 %**	>=50%	53	Reference
	1–49%	149	1.40 (0.96, 2.06) 0.083
	<1%	359	1.79 (1.25, 2.56) **0.001**
**Line of therapy**	1	549	Reference
	>=2	12	0.98 (0.53, 1.82) 0.948
**Antibiotics**	No Antibiotics	353	Reference
	Antibiotics	208	1.40 (1.15, 1.70) **<0.001**
**Liver metastases**	Absence	496	Reference
	Presence	65	1.56 (1.18, 2.05) **0.002**
**Brain metastases**	Absence	431	Reference
	Presence	130	1.06 (0.86, 1.32) 0.580

E.

Variable		N	p
**Age**		562	1.01 (1.00, 1.02) 0.26
**Sex**	F	259	Reference
	M	303	1.08 (0.87, 1.34) 0.49
**Histology**	Adenocarcinoma	449	Reference
	Squamous cell carcinoma	73	1.02 (0.74, 1.41) 0.90
	Other	40	1.56 (1.03, 2.35) **0.04**
**ECOG PS**	0–1	494	Reference
	>=2	68	1.05 (0.76, 1.46) 0.77
**Smoking**	Current/Former	485	Reference
	Never	77	1.12 (0.83, 1.50) 0.47
**PD-L1 %**	>=50%	53	Reference
	1–49%	149	1.19 (0.76, 1.87) 0.44
	<1%	360	1.47 (0.97, 2.23) 0.07
**Line of therapy**	1	550	Reference
	>=2	12	0.65 (0.30, 1.41) 0.28
**Antibiotics**	No Antibiotics	354	Reference
	Antibiotics	208	1.76 (1.41, 2.19) **<0.001**
**Liver metastases**	Absence	497	Reference
	Presence	65	1.83 (1.36, 2.47) **<0.001**
**Brain metastases**	Absence	431	Reference
	Presence	131	1.18 (0.92, 1.51) 0.19

Supplementary Figure 4. Association between antibiotic exposure and outcomes to combination platinum-doublet chemotherapy and immunotherapy in patients with non-small cell lung cancer -60+42d window. A. Objective response rate, **B.** Progression-free survival, **C.** Overall survival in antibiotics vs. no antibiotics group. **D.** Multivariable cox model for PFS and **E.** OS for antibiotics vs. no antibiotics while adjusting for standard prognostic features in non-small cell lung cancer. mPFS, median progression-free survival; mOS, median overall survival; HR, hazard ratio; 95%CI, 95 % confidence interval. Median survival times given and numbers in parentheses represent 95% confidence intervals.

Supplementary Figure 5

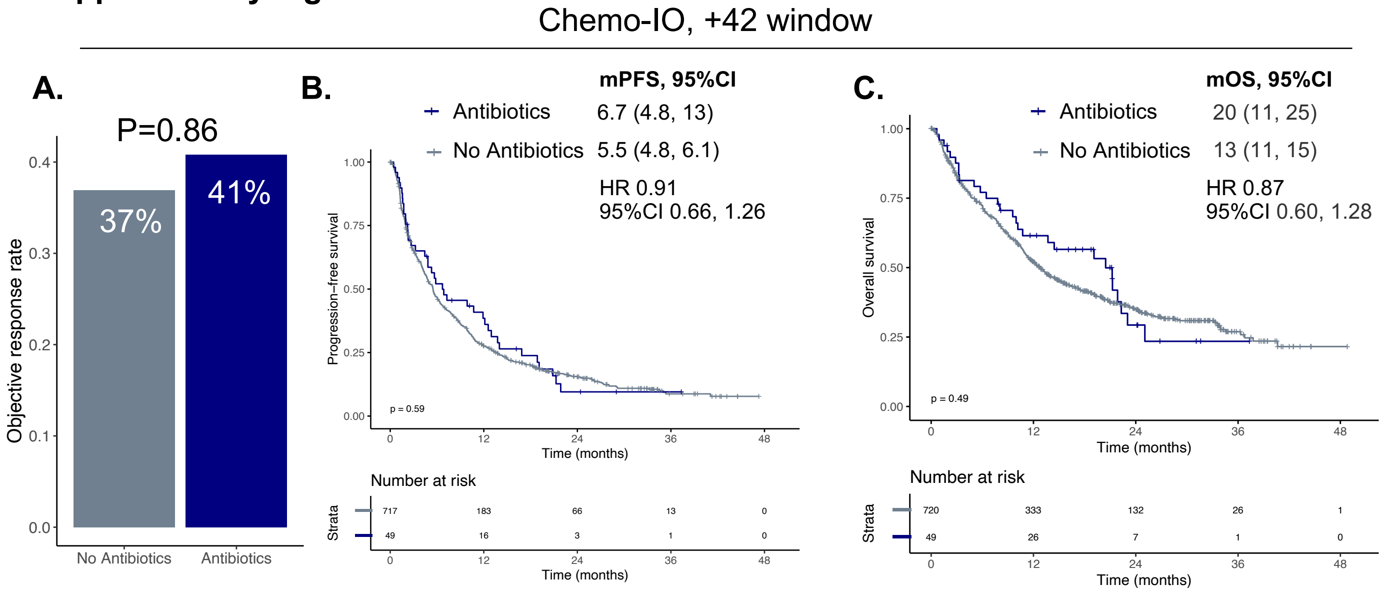

Supplementary Figure 5. Association between antibiotic exposure and outcomes to Chemo-IO in patients with non-small cell lung cancer +42d window. A. Objective response rate, **B.** Progression-free survival, **C.** Overall survival in antibiotics vs. no antibiotics group. mPFS, median progression-free survival; mOS, median overall survival; HR, hazard ratio; 95%CI, 95 % confidence interval. Median survival times given and numbers in parentheses represent 95% confidence intervals.

Supplementary Figure 6

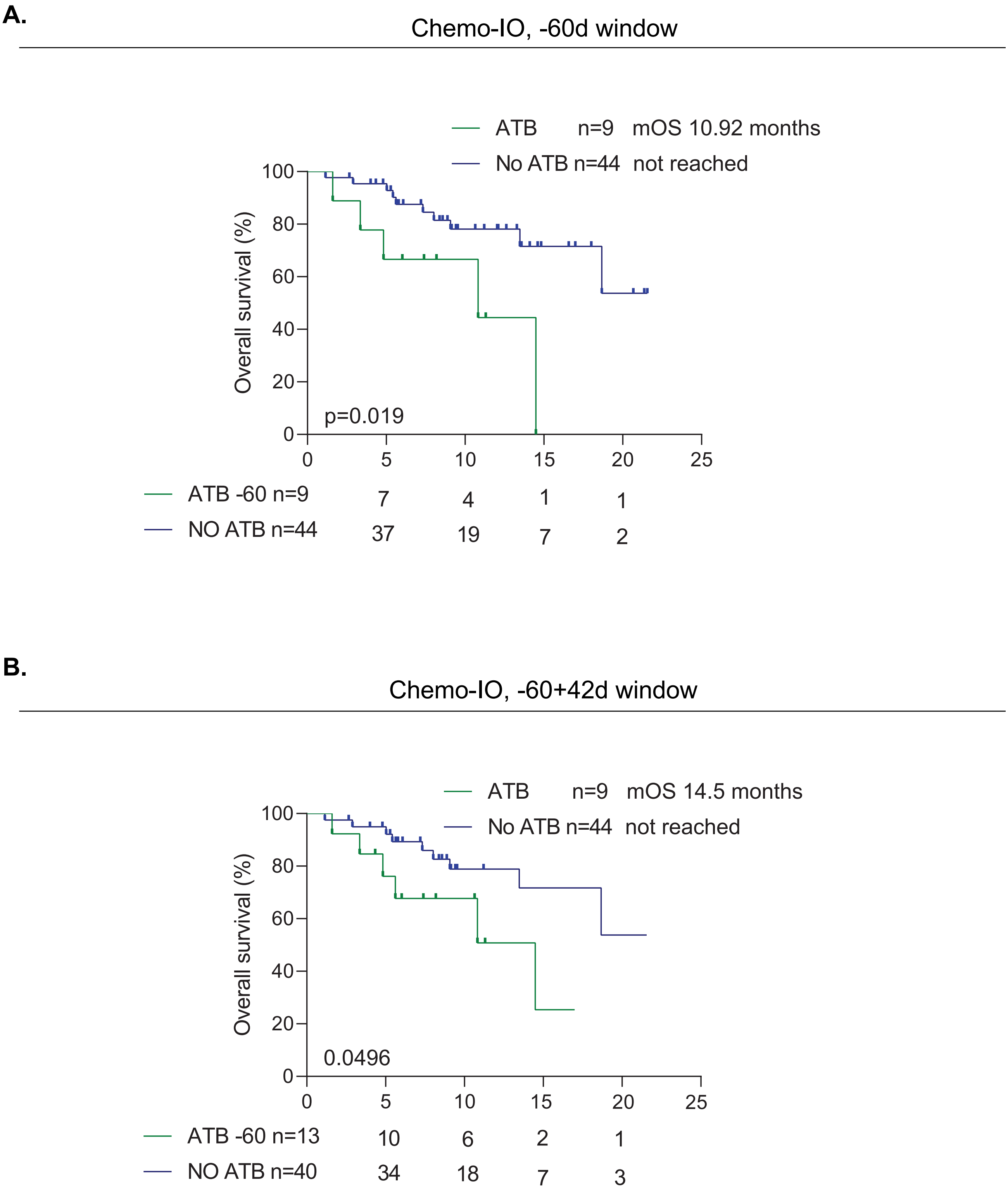

Supplementary Figure 6. Association between antibiotic exposure and survival to combination platinum-doublet chemotherapy and immunotherapy in patients with non-small cell lung cancer in the IMMUNOLIFE Cohort. A. Overall survival in antibiotics vs. no antibiotics group (-60d window). **B.** Overall survival in antibiotics vs. no antibiotics group (-60d+42d window); mOS, median overall survival.

Supplementary Figure 7

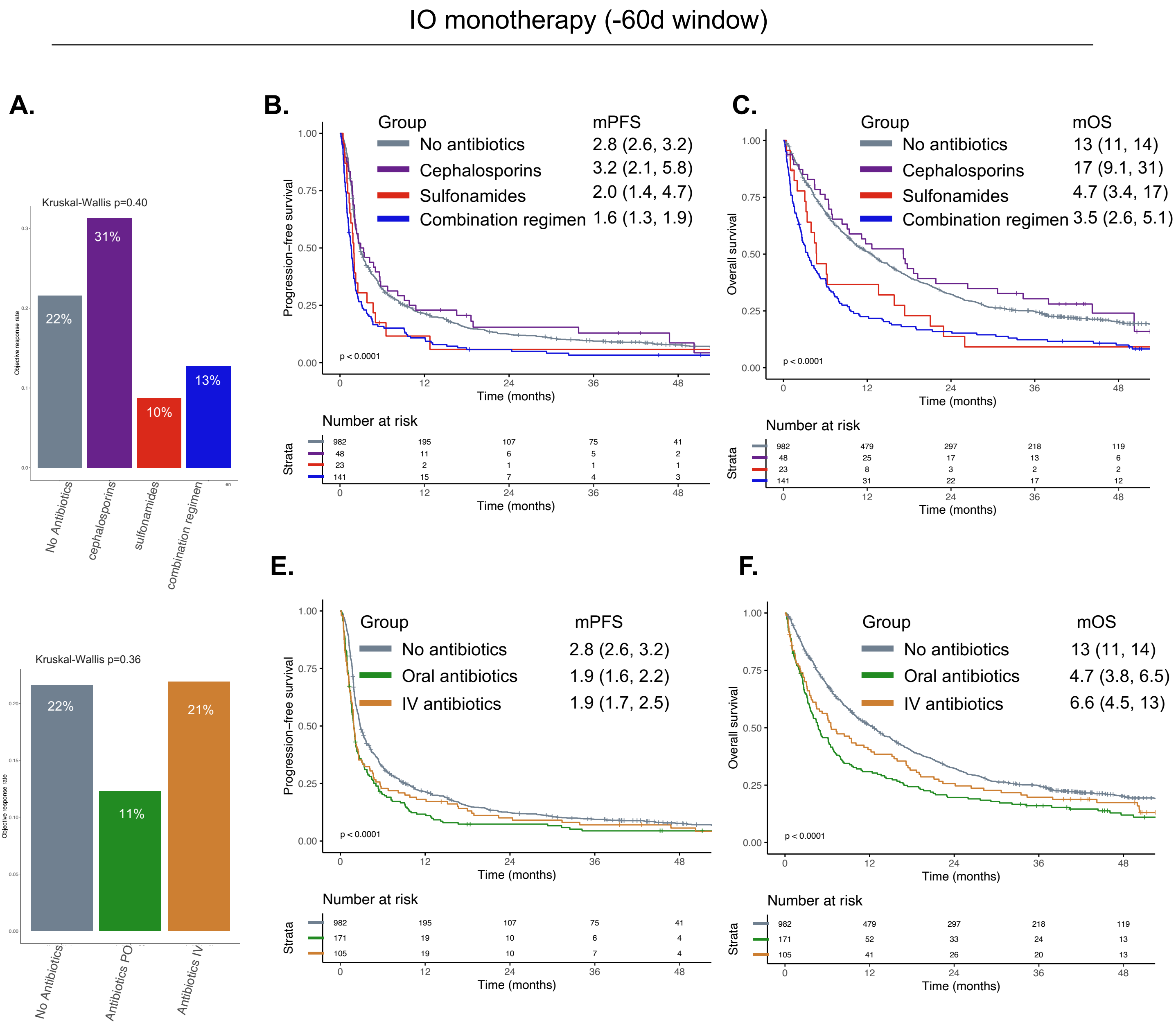

Supplementary Figure 7. Association between antibiotic class and route of administration with outcomes to IO monotherapy in patients with non-small cell lung cancer. A. Objective response rate, **B.** Progression-free survival, **C.** Overall survival according to antibiotic class. **D.** Objective response rate, **E.** Progression-free survival, **F.** Overall survival according to antibiotic route. mPFS, median progression-free survival; mOS, median overall survival; HR, hazard ratio; 95%CI, 95 % confidence interval. Median survival times given and numbers in parentheses represent 95% confidence intervals.

Supplementary Figure 8

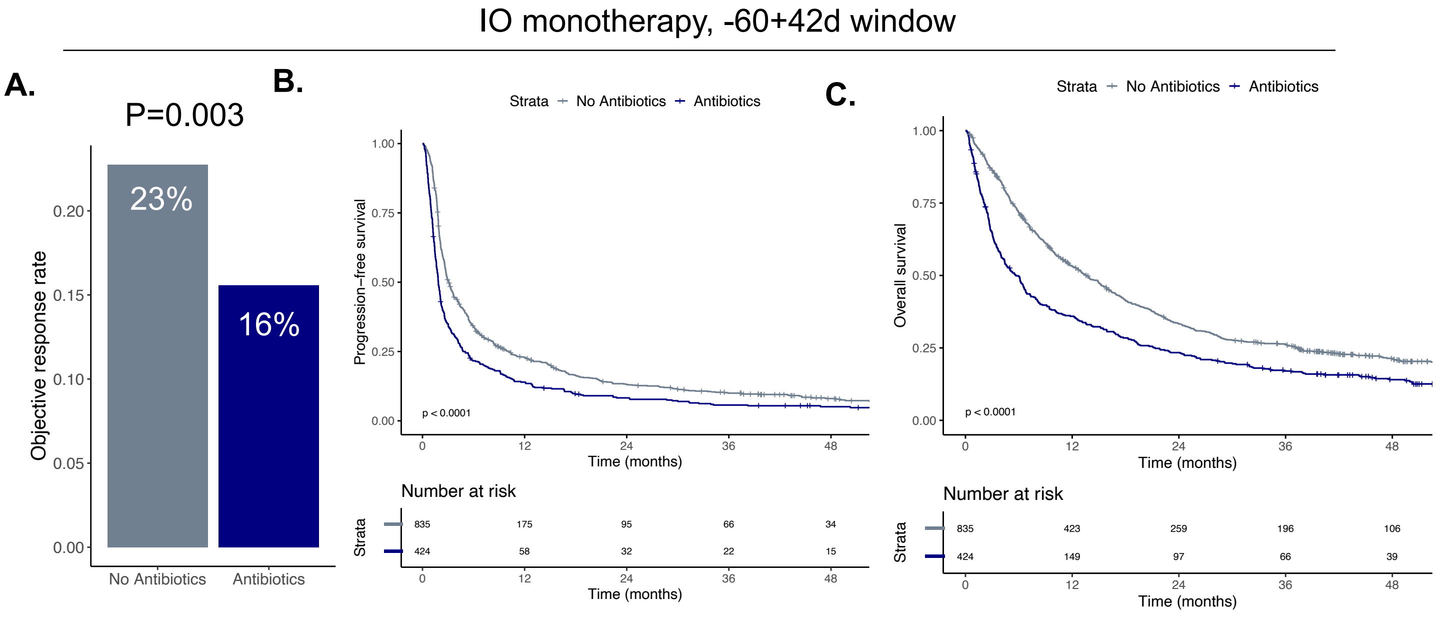

D.

Variable		N	Hazard ratio		p
**Age**		457		1.00 (0.99, 1.01)	0.478
**Sex**	F	245		Reference	
	M	212		0.98 (0.80, 1.21)	0.853
**Histology**	Adenocarcinoma	340		Reference	
	Squamous cell carcinoma	75		1.44 (1.08, 1.92)	**0.012**
	Other	42		0.75 (0.51, 1.11)	0.152
**ECOG PS**	0–1	409		Reference	
	>=2	48		1.31 (0.95, 1.81)	0.094
**Smoking**	Current/Former	415		Reference	
	Never	42		1.62 (1.15, 2.28)	**0.006**
**PD-L1%**	>=50%	295		Reference	
	1–49%	54		1.13 (0.81, 1.56)	0.474
	<1%	108		1.67 (1.30, 2.15)	**<0.001**
**Line of therapy**	1	251		Reference	
	>=2	206		1.17 (0.94, 1.46)	0.169
**Antibiotics**	No Antibiotics	288		Reference	
	Antibiotics	169		1.26 (1.02, 1.54)	**0.031**
**Liver metastases**	Absence	351		Reference	
	Presence	106		1.19 (0.95, 1.51)	0.135
**Brain metastases**	Absence	343		Reference	
	Presence	114		1.13 (0.89, 1.43)	0.318

1 1.5 2

PFS better PFS worse

E.

Variable		N	Hazard ratio		p
**Age**		457		1.02 (1.01, 1.03)	**0.001**
**Sex**	F	245		Reference	
	M	212		0.96 (0.76, 1.20)	0.707
**Histology**	Adenocarcinoma	340		Reference	
	Squamous cell carcinoma	75		1.46 (1.07, 1.99)	**0.018**
	Other	42		1.01 (0.68, 1.49)	0.979
**ECOG PS**	0–1	409		Reference	
	>=2	48		1.38 (0.96, 1.96)	0.080
**Smoking**	Current/Former	415		Reference	
	Never	42		1.09 (0.74, 1.60)	0.679
**PD-L1%**	>=50%	295		Reference	
	1–49%	54		1.16 (0.80, 1.66)	0.437
	<1%	108		1.77 (1.34, 2.33)	**<0.001**
**Line of therapy**	1	251		Reference	
	>=2	206		1.11 (0.87, 1.41)	0.405
**Antibiotics**	No Antibiotics	288		Reference	
	Antibiotics	169		1.38 (1.10, 1.72)	**0.005**
**Liver metastases**	Absence	351		Reference	
	Presence	106		1.32 (1.03, 1.70)	0.028
**Brain metastases**	Absence	343		Reference	
	Presence	114		1.28 (0.99, 1.66)	0.057

1 1.5 2

OS better OS worse

Supplementary Figure 8. Association between antibiotic exposure and outcomes to monotherapy immunotherapy in patients with non-small cell lung cancer -60+42d window. A. Objective response rate, **B.** Progression-free survival, **C.** Overall survival in antibiotics vs. no antibiotics group. **D.** Multivariable cox model for PFS and **E.** OS for antibiotics vs. no antibiotics while adjusting for standard prognostic features in non-small cell lung cancer. mPFS, median progression-free survival; mOS, median overall survival; HR, hazard ratio; 95%CI, 95 % confidence interval. Median survival times given and numbers in parentheses represent 95% confidence intervals.

Supplementary Figure 9

NSCLC

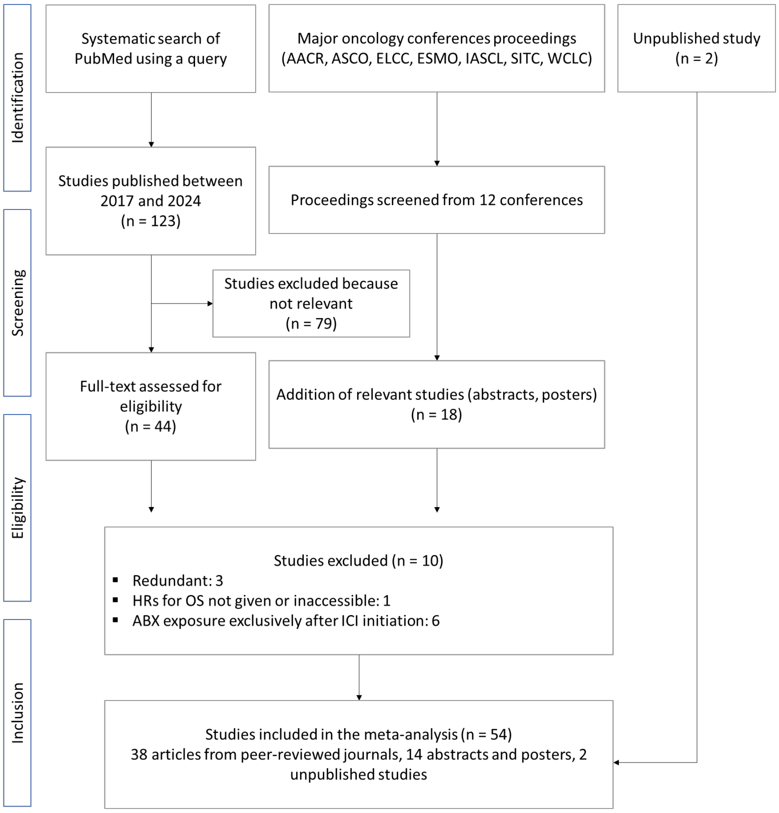

All tumors

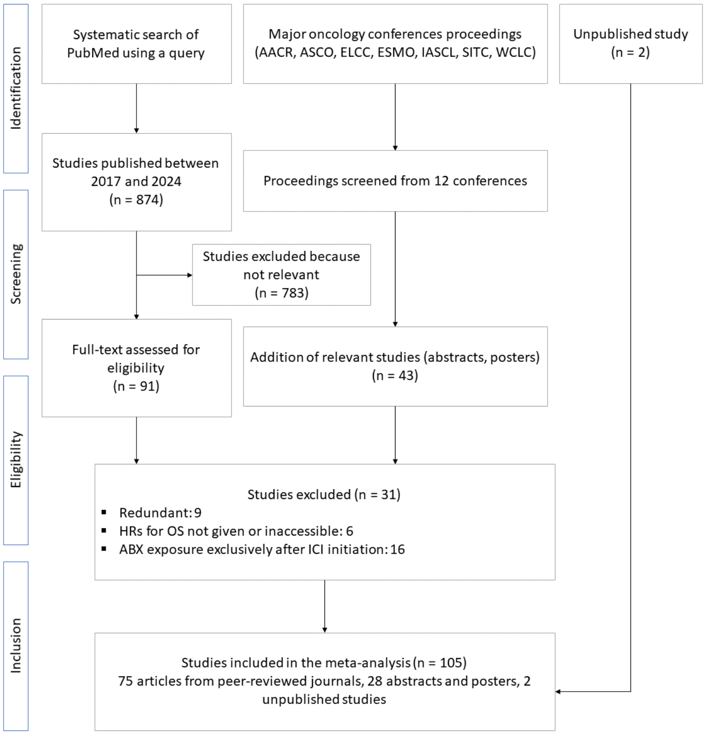

Supplementary Figure 9. Study selection for meta-analysis. A. NSCLC; Non-small cell lung cancer. B. All tumors

Supplementary Figure 10

NSCLC meta-analysis (before and during IO) (-60+42d)

Study	ATB (N=3777)	Total (N=12220)	Treatment	Weight	HR [95%CI]
Manning−Bennett et al. 2023	473	755	IO	3.98%	1.29 [1.04, 1.59]
Elkrief et al. 2023	277	1259	IO	4.27%	1.47 [1.30, 1.70]
Chalabi et al. 2020	169	757	IO	3.97%	1.32 [1.06, 1.63]
Metges et al. 2018	153	325	IO	3.40%	0.67 [0.48, 0.94]
Riudavets et al. 2019	141	267	IO	3.67%	0.98 [0.74, 1.30]
Ochi et al. 2021	98	531	IO	3.63%	1.38 [1.03, 1.84]
Do et al. 2018	87	109	IO	1.96%	3.45 [1.72, 6.67]
Qiu et al. 2022	80	148	IO	2.71%	2.28 [1.41, 3.70]
Spakowicz et al. 2020	70	196	IO	3.41%	1.50 [1.07, 2.09]
Geum et al. 2021	70	140	IO	1.96%	2.29 [1.16, 4.51]
Huemer et al. 2019	62	142	IO	2.74%	0.90 [0.56, 1.45]
Vihinen et al. 2023	61	199	IO	3.33%	1.86 [1.31, 2.65]
Bagley et al. 2019	61	1960	IO	1.70%	1.16 [0.54, 2.48]
Mielgo Rubio et al. 2018 − Oral ATB	56	168	IO	2.87%	1.16 [0.74, 1.81]
von Itzstein et al. 2022	54	133	IO	3.04%	1.83 [1.21, 2.76]
Kulkarni et al. 2020	54	140	IO	1.48%	2.18 [0.93, 5.09]
Ahmed Y et al. 2020	53	141	IO	2.85%	2.63 [1.67, 4.13]
Nyein et al. 2022	46	256	IO	3.10%	1.35 [0.91, 2.02]
Peng et al. 2022	41	117	IO	2.52%	1.42 [0.84, 2.41]
Kostine et al. 2021	41	150	IO	3.00%	1.54 [1.01, 2.34]
Hamada et al. 2021	38	69	IO	1.74%	1.99 [0.91, 4.09]
Mielgo Rubio et al. 2019 − IV ATB	36	121	IO	1.64%	3.75 [1.71, 8.21]
Verschueren et al. 2021	35	221	IO	2.97%	1.20 [0.79, 1.85]
Rounis et al. 2021	34	66	IO	2.32%	1.35 [0.76, 2.41]
Forde et al. 2020	34	86	IO	1.64%	1.95 [0.89, 4.28]
Ruiz−Patino et al. 2020	32	140	IO	2.92%	2.88 [1.86, 4.45]
Ouaknine et al. 2019	30	72	IO	1.78%	2.20 [1.10, 4.80]
Svaton et al. 2020	27	224	IO	1.73%	0.66 [0.31, 1.40]
Ren et al. 2021	27	98	IO	1.63%	1.60 [0.74, 3.60]
Galli et al. 2019	27	157	IO	3.21%	1.22 [0.84, 1.78]
Mielgo Rubio et al. 2018 − IV ATB	24	168	IO	2.42%	2.85 [1.64, 4.95]
Zhao et al. 2019	20	109	IO	2.03%	3.45 [1.79, 6.67]
Huemer et al. 2019	20	43	IO	1.34%	2.04 [1.02, 6.38]
Castello et al. 2021	20	50	IO	0.31%	1.60 [0.18, 14.01]
Mielgo Rubio et al. 2019 − Oral ATB	19	121	IO	1.00%	1.00 [0.33, 3.05]
Hossain et al. 2020	18	63	IO	1.77%	2.01 [0.96, 4.20]
Castro−Balado et al. 2021	17	49	IO	1.23%	3.80 [1.44, 10.05]
Manning−Bennett et al. 2023	976	1937	Chemo+IO	3.92%	1.23 [0.98, 1.54]
Elkrief et al. 2023	183	769	Chemo+IO	4.02%	1.50 [1.20, 1.80]
Derosa et al. *(unpublished)*	13	53	Chemo+IO	0.81%	2.67 [0.75, 9.48]
RE Model (Chi^2 = 101.66, df = 39, $p < 0.001$; I^2 = 70.6%)					1.58 [1.39, 1.79]

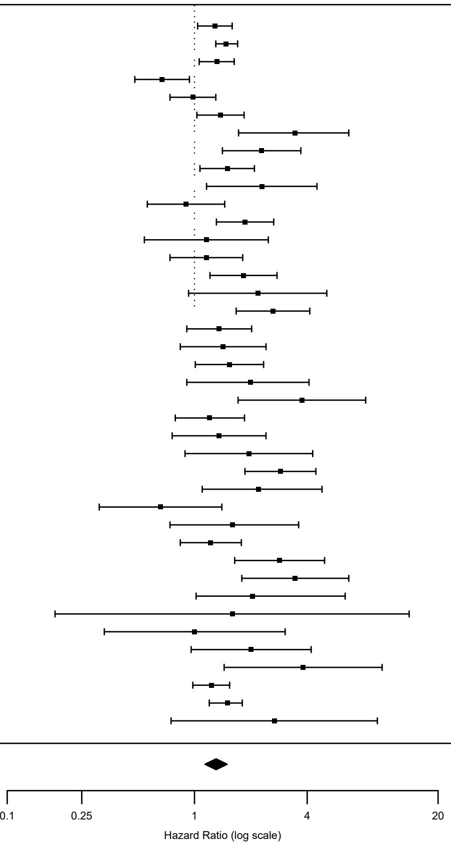

Supplementary Figure 10. Meta-analysis of all studies examining the association of antibiotic exposure on overall survival to Immunotherapy (IO) alone or platinum doublet Chemo-IO in non-small cell lung cancer before and during therapy in patients with NSCLC [-60; +42 day window].

Supplementary Figure 11

All solid tumors meta-analysis (strictly before IO) (-60d)

Study	ATB (N=4522)	Total (N=17452)	Cancer type	Treatment	Weight	HR [95%CI]
Eng et al. 2023	1627	2737	Aggregated	IO	3.84%	1.19 [1.09, 1.30]
Stokes et al. 2021	762	3634	NSCLC	IO	3.83%	1.31 [1.20, 1.44]
Elkrief et al. 2023	277	1259	NSCLC	IO	3.76%	1.47 [1.30, 1.70]
Hopkins et al. 2022	194	2723	NSCLC	IO	3.59%	1.23 [1.00, 1.51]
Cortellini et al. 2021	131	950	NSCLC	IO	3.52%	1.42 [1.13, 1.79]
Lu et al. 2020	129	340	NSCLC	IO	1.30%	2.90 [1.10, 8.10]
Jung M et al. 2021	114	228	Gastric	IO	3.33%	2.47 [1.85, 3.31]
Kim H et al. 2019	108	234	Aggregated	IO	3.15%	1.78 [1.26, 2.52]
Derosa et al. 2022	69	338	NSCLC	IO	3.10%	1.36 [0.95, 1.94]
Kim CG et al. 2023	67	152	Gastric	IO	3.14%	2.29 [1.62, 3.24]
Greally et al. 2019	62	161	Gastric	IO	3.07%	1.26 [0.87, 1.81]
Kim H et al. 2019	60	131	NSCLC	IO	2.75%	2.48 [1.57, 3.91]
Kim CG et al. 2023	57	123	Gastric	IO	3.26%	3.51 [2.89, 5.38]
Ernst et al. 2021	56	427	RCC	IO	2.60%	1.10 [0.67, 1.81]
Derosa et al. 2018	48	239	NSCLC	IO	2.47%	4.40 [2.60, 7.60]
Jung HJ et al. 2023	47	215	Gynecologic	IO	2.88%	2.42 [1.59, 3.68]
Tomita et al. 2023	46	100	NSCLC	IO	2.47%	1.40 [0.82, 2.40]
Facchinetti et al. 2020	36	153	NSCLC	IO	3.14%	2.00 [1.25, 2.50]
Araujo et al. 2021	34	216	Aggregated	IO	2.94%	2.39 [1.60, 3.58]
Giordan et al. 2021	31	212	Aggregated	IO	1.73%	2.68 [1.22, 5.92]
Swami et al. 2020	30	169	MM	IO	2.43%	1.73 [1.00, 2.99]
Pinato et al. 2019	29	119	NSCLC	IO	1.85%	9.30 [4.42, 19.55]
Buti et al. 2020	27	217	Aggregated	IO	2.75%	2.07 [1.31, 3.25]
Jun et al. 2020	25	95	HCC	IO	2.24%	1.36 [0.74, 2.49]
Vitorino et al. 2021	24	114	Aggregated	IO	2.19%	0.97 [0.52, 1.81]
Chambers et al. 2021	23	101	Gynecologic	IO	2.16%	2.29 [1.22, 4.32]
Sen et al. 2018	19	172	Aggregated	IO	2.57%	2.00 [1.20, 3.30]
Thompson et al. 2017	18	74	NSCLC	IO	1.58%	3.50 [1.49, 8.22]
Pinato et al. 2019	17	38	MM	IO	0.75%	7.50 [1.77, 31.71]
Derosa et al. 2018	16	121	RCC	IO	1.08%	3.50 [1.10, 10.80]
Akashi et al. 2023	16	41	UC	IO	1.66%	1.68 [0.74, 3.80]
Kim JH et al. 2022	15	60	Gastric	IO	1.93%	4.04 [1.98, 8.24]
Greally et al. 2019	14	161	Gastric	IO	2.30%	2.40 [1.30, 4.20]
Hakozaki et al. 2019	13	90	NSCLC	IO	1.20%	2.02 [0.70, 5.83]
Ruiz−Banobre et al. 2021	11	119	UC	IO	1.76%	1.29 [0.59, 2.80]
Elkrief et al. 2019	10	74	MM	IO	0.97%	3.70 [1.08, 12.50]
Pinato et al. 2019	6	39	Aggregated	IO	1.04%	7.80 [2.42, 25.17]
Shen et al. 2021	4	36	HCC	IO	0.87%	3.15 [0.85, 11.71]
Elkrief et al. 2023	183	769	NSCLC	Chemo+IO	3.60%	1.50 [1.20, 1.80]
Cortellini et al. 2021	47	302	NSCLC	Chemo+IO	2.78%	1.42 [0.91, 2.22]
Clark et al. 2020	11	77	NSCLC	Chemo+IO	1.69%	2.30 [1.10, 5.50]
Derosa et al. *(unpublished)*	9	53	NSCLC	Chemo+IO	0.71%	3.30 [0.74, 14.78]
RE Model (Chi^2 = 189.22, df = 41, p < 0.001; I^2 = 84.1%)						1.98 [1.72, 2.28]

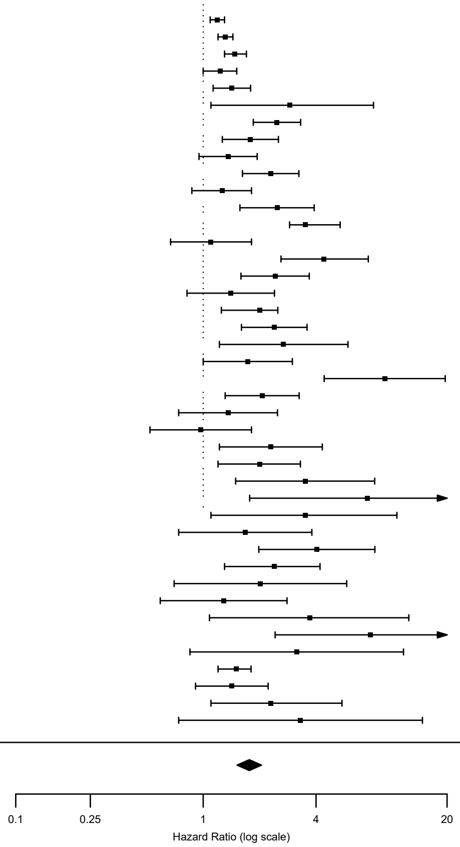

Supplementary Figure 11. Meta-analysis of all studies examining the association of antibiotic exposure on overall survival to Immunotherapy (IO) alone or platinum doublet Chemo-IO in all solid tumors strictly before therapy [-60 day window].

Supplementary Figure 12

All solid tumors meta-analysis before and during IO (-60+42d)

Study	ATB (N=7340)	Total (N=26603)	Cancer Type	Treatment	Weight	HR [95%CI]
Abu−Sbeih et al. 2019	569	826	Aggregated	IO	1.87%	2.21 [1.70, 2.86]
Weinstock et al. 2020	482	1747	UC	IO	2.29%	1.80 [1.60, 2.00]
Manning−Bennett et al. 2023	473	755	NSCLC	IO	2.02%	1.29 [1.04, 1.59]
Vellanki et al. 2020	372	1037	H&N	IO	2.12%	1.70 [1.42, 2.03]
Elkrief et al. 2023	277	1259	NSCLC	IO	2.24%	1.47 [1.30, 1.70]
Hopkins et al. 2020	235	896	UC	IO	2.10%	1.44 [1.19, 1.73]
Chalabi et al. 2020	169	757	NSCLC	IO	2.02%	1.32 [1.06, 1.63]
Fessas et al. 2021	155	402	HCC	IO	1.79%	1.01 [0.76, 1.35]
Metges et al. 2018	153	325	NSCLC	IO	1.63%	0.67 [0.48, 0.94]
Riudavets et al. 2019	141	267	NSCLC	IO	1.81%	0.98 [0.74, 1.30]
Cren et al. 2020	117	1585	MM	IO	1.89%	1.88 [1.46, 2.43]
Gaucher et al. 2021	112	372	Aggregated	IO	1.83%	1.75 [1.33, 2.30]
Cheung et al. 2021	109	395	HCC	IO	1.59%	1.81 [1.28, 2.57]
Derosa et al. 2021	104	707	RCC	IO	1.85%	1.59 [1.22, 2.09]
Ochi et al. 2021	98	531	NSCLC	IO	1.78%	1.38 [1.03, 1.84]
Tinsley et al. 2019	92	291	Aggregated	IO	1.57%	1.47 [1.03, 2.10]
Spakowicz et al. 2020	89	225	Aggregated	IO	1.62%	2.11 [1.50, 2.97]
Qiu et al. 2022	80	148	NSCLC	IO	1.22%	2.28 [1.41, 3.70]
Spakowicz et al. 2020	74	321	MM	IO	1.62%	1.46 [1.04, 2.06]
Vihinen et al. 2023	71	222	MM	IO	1.46%	1.53 [1.03, 2.26]
Okuyama et al. 2022	71	155	UC	IO	1.22%	1.02 [0.63, 1.65]
Spakowicz et al. 2020	70	196	NSCLC	IO	1.64%	1.50 [1.07, 2.09]
Khan M. et al. 2020	70	130	UC	IO	0.64%	1.46 [0.65, 3.28]
Geum et al. 2021	70	140	NSCLC	IO	0.82%	2.29 [1.16, 4.51]
Huemer et al. 2019	62	142	NSCLC	IO	1.23%	0.90 [0.56, 1.45]
Vihinen et al. 2023	61	199	NSCLC	IO	1.58%	1.86 [1.31, 2.65]
Fukuokaya et al. 2022	61	227	UC	IO	1.01%	1.33 [0.75, 2.36]
Bagley et al. 2019	61	1960	NSCLC	IO	0.70%	1.16 [0.54, 2.48]
Iglesias−Santamaria et al. 2020	60	102	Aggregated	IO	1.05%	1.53 [0.88, 2.66]
Mielgo Rubio et al. 2018 − Oral ATB	56	168	NSCLC	IO	1.31%	1.16 [0.74, 1.81]
von Itzstein et al. 2022	54	133	NSCLC	IO	1.41%	1.83 [1.21, 2.76]
Kulkarni et al. 2020	54	140	NSCLC	IO	0.60%	2.18 [0.93, 5.09]
Perez−Ruiz et al. 2020	53	253	Aggregated	IO	1.78%	2.34 [1.75, 3.13]
Ahmed Y et al. 2020	53	141	NSCLC	IO	1.29%	2.63 [1.67, 4.13]
Nyein et al. 2022	46	256	NSCLC	IO	1.44%	1.35 [0.91, 2.02]
Kostine et al. 2021	42	293	MM	IO	1.50%	1.80 [1.23, 2.63]
Zhong et al. 2024	41	214	SCLC	IO	1.53%	1.46 [1.01, 2.11]
Peng et al. 2022	41	117	NSCLC	IO	1.11%	1.42 [0.84, 2.41]
Kostine et al. 2021	41	150	NSCLC	IO	1.38%	1.54 [1.01, 2.34]
Ren et al. 2021	38	143	UC	IO	1.06%	2.30 [1.30, 3.90]
Hamada et al. 2021	38	69	NSCLC	IO	0.71%	1.99 [0.91, 4.09]
Routy et al. 2018	37	140	Aggregated	IO	1.11%	2.21 [1.30, 3.74]
Ren et al. 2021	36	317	HCC	IO	1.22%	1.80 [1.11, 2.92]
Mielgo Rubio et al. 2019 − IV ATB	36	121	NSCLC	IO	0.67%	3.75 [1.71, 8.21]
Verschueren et al. 2021	35	221	NSCLC	IO	1.37%	1.20 [0.79, 1.85]
Spakowicz et al. 2020	34	104	RCC	IO	1.13%	2.01 [1.20, 3.38]
Rounis et al. 2021	34	66	NSCLC	IO	1.00%	1.35 [0.76, 2.41]
Forde et al. 2020	34	86	NSCLC	IO	0.67%	1.95 [0.89, 4.28]
Pinato et al. 2022	33	258	HCC	IO	1.91%	1.31 [1.02, 1.68]
Kapoor et al. 2020	33	155	Aggregated	IO	2.40%	1.02 [1.00, 1.04]
Guven et al. 2021	33	93	RCC	IO	0.80%	2.30 [1.15, 4.60]
Ruiz−Patino et al. 2020	32	140	NSCLC	IO	1.34%	2.88 [1.86, 4.45]
Lalani et al. 2020	31	146	RCC	IO	0.86%	1.44 [0.75, 2.77]
Ouaknine et al. 2019	30	72	NSCLC	IO	0.73%	2.20 [1.10, 4.80]
Svaton et al. 2020	27	224	NSCLC	IO	0.71%	0.66 [0.31, 1.40]
Ren et al. 2021	27	98	NSCLC	IO	0.66%	1.60 [0.74, 3.60]
Galli et al. 2019	27	157	NSCLC	IO	1.51%	1.22 [0.84, 1.78]
Agarwal et al. 2019	26	101	UC	IO	0.99%	2.01 [1.12, 3.62]
Wada et al. 2023	24	110	Aggregated	IO	0.78%	1.56 [0.77, 3.17]
Mielgo Rubio et al. 2018 − IV ATB	24	168	NSCLC	IO	1.06%	2.85 [1.64, 4.95]
Kulkarni et al. 2020	24	55	RCC	IO	0.43%	4.20 [1.47, 11.98]
Plana et al. 2020	23	74	H&N	IO	0.88%	1.05 [0.55, 2.00]
Bagley et al. 2019	23	1177	MM	IO	0.26%	0.70 [0.17, 2.85]
Guo et al. 2019	21	49	Gastric	IO	0.61%	5.88 [2.55, 13.55]
Zhao et al. 2019	20	109	NSCLC	IO	0.86%	3.45 [1.79, 6.67]
Spakowicz et al. 2020	20	38	UC	IO	0.71%	3.01 [1.42, 6.40]
Kostine et al. 2021	20	83	RCC	IO	0.89%	1.98 [1.05, 3.74]
Huemer et al. 2019	20	43	NSCLC	IO	0.53%	2.04 [1.02, 6.38]
Castello et al. 2021	20	50	NSCLC	IO	0.12%	1.60 [0.18, 14.01]
Spakowicz et al. 2020	19	64	H&N	IO	0.79%	1.37 [0.68, 2.75]
Mielgo Rubio et al. 2019 − Oral ATB	19	121	NSCLC	IO	0.39%	1.00 [0.33, 3.05]
Hossain et al. 2020	18	63	NSCLC	IO	0.73%	2.01 [0.96, 4.20]
Castro−Balado et al. 2021	17	49	NSCLC	IO	0.49%	3.80 [1.44, 10.05]
Ahmed J et al. 2018	17	60	Aggregated	IO	0.46%	2.90 [1.10, 8.10]
Ishiyama et al. 2021	15	67	UC	IO	0.69%	3.34 [1.55, 7.20]
Spakowicz et al. 2020	14	45	Sarcoma	IO	0.56%	1.16 [0.48, 2.82]
Ren et al. 2021	14	46	Gastric	IO	0.59%	3.00 [1.30, 7.20]
Spahn et al. 2020	13	99	HCC	IO	0.74%	1.76 [0.84, 3.66]
Tomisaki et al. 2022	12	40	UC	IO	0.29%	0.76 [0.20, 2.85]
Ng Wei Qi et al. 2021	9	44	Aggregated	IO	0.15%	4.90 [0.73, 33.05]
Manning−Bennett et al. 2023	976	1937	NSCLC	Chemo+IO	1.98%	1.23 [0.98, 1.54]
Elkrief et al. 2023	183	769	NSCLC	Chemo+IO	2.05%	1.50 [1.20, 1.80]
Pinato et al. 2022	102	584	HCC	Chemo+IO	2.21%	1.14 [0.99, 1.32]
Derosa et al. *(unpublished)*	13	53	NSCLC	Chemo+IO	0.31%	2.67 [0.75, 9.48]
RE Model (Chi^2 = 552.15, df = 83, p < 0.001; I^2 = 77.7%)						1.60 [1.49, 1.73]

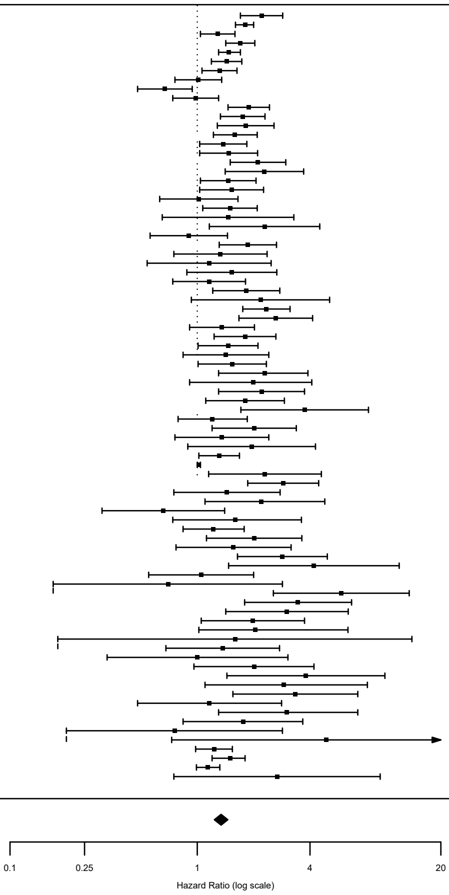

Supplementary Figure 12. Meta-analysis of all studies examining the association of antibiotic exposure on overall survival to Immunotherapy (IO) alone or platinum doublet Chemo-IO in all solid tumors strictly before and during IO therapy [-60; +42 day window]. .

Supplementary Figure 13

All solid tumors meta-analysis before and during IO (-90+90d)

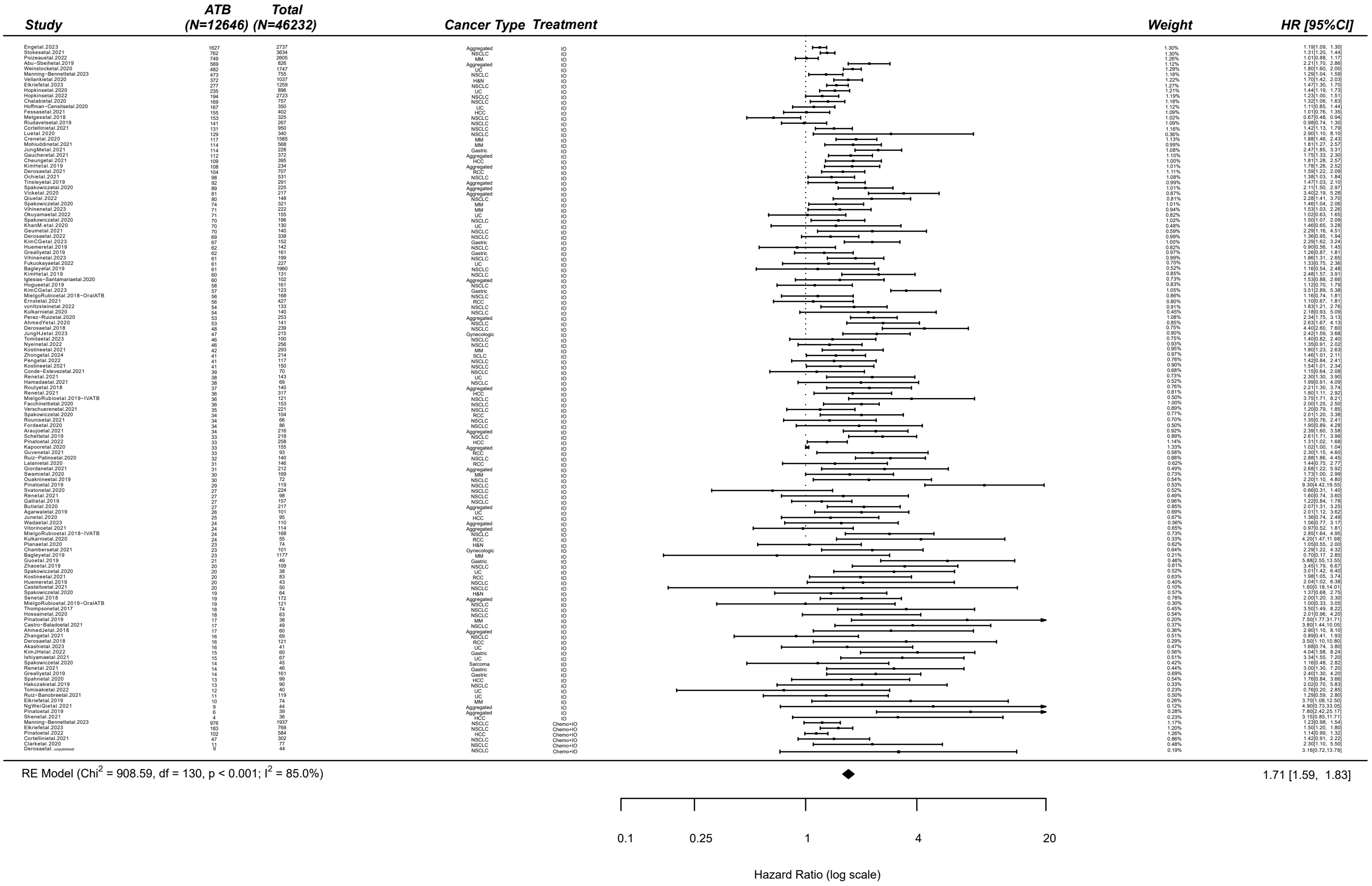

Supplementary Figure 13. Meta-analysis of all studies examining the association of antibiotic exposure on overall survival to Immunotherapy (IO) alone or platinum doublet Chemo-IO in all solid tumors strictly before and during IO therapy [-90; +90 day window].
